# Phylogeny and evolution of Rab7 and Rab9 proteins

**DOI:** 10.1186/1471-2148-9-101

**Published:** 2009-05-14

**Authors:** Paweł Mackiewicz, Elżbieta Wyroba

**Affiliations:** 1 University of Wrocław, Faculty of Biotechnology, Department of Genomics, 63/77 Przybyszewskiego Street, 51-148 Wrocław, Poland; 2 Department of Cell Biology, Nencki Institute of Experimental Biology, 3 Pasteur Street, 02-093 Warsaw, Poland

## Abstract

**Background:**

An important role in the evolution of intracellular trafficking machinery in eukaryotes played small GTPases belonging to the Rab family known as pivotal regulators of vesicle docking, fusion and transport. The Rab family is very diversified and divided into several specialized subfamilies. We focused on the VII functional group comprising Rab7 and Rab9, two related subfamilies, and analysed 210 sequences of these proteins. Rab7 regulates traffic from early to late endosomes and from late endosome to vacuole/lysosome, whereas Rab9 participates in transport from late endosomes to the trans-Golgi network.

**Results:**

Although Rab7 and Rab9 proteins are quite small and show heterogeneous rates of substitution in different lineages, we found a phylogenetic signal and inferred evolutionary relationships between them. Rab7 proteins evolved before radiation of main eukaryotic supergroups while Rab9 GTPases diverged from Rab7 before split of choanoflagellates and metazoans. Additional duplication of Rab9 and Rab7 proteins resulting in several isoforms occurred in the early evolution of vertebrates and next in teleost fishes and tetrapods. Three Rab7 lineages emerged before divergence of monocots and eudicots and subsequent duplications of Rab7 genes occurred in particular angiosperm clades. Interestingly, several Rab7 copies were identified in some representatives of excavates, ciliates and amoebozoans. The presence of many Rab copies is correlated with significant differences in their expression level. The diversification of analysed Rab subfamilies is also manifested by non-conserved sequences and structural features, many of which are involved in the interaction with regulators and effectors. Individual sites discriminating different subgroups of Rab7 and Rab9 GTPases have been identified.

**Conclusion**

Phylogenetic reconstructions of Rab7 and Rab9 proteins were performed by a variety of methods. These Rab GTPases show diversification both at the phylogenetic, expression and structural levels. The presence of many Rab7 and Rab9 isoforms suggests their functional specialization and complexity of subcellular trafficking even in unicellular eukaryotes. The identified less conserved regions in analysed Rab sequences may directly contribute to such a differentiation.

## Background

The origin of eukaryotic cells was one of the major evolutionary transitions, although very interesting and challenging, still remains poorly understood. The development of the endomembrane system and cellular trafficking machinery are crucial stage in eukaryotic cell evolution [1-3]. An important role in this evolution was played by small GTPases belonging to the Ras superfamily [1,2] that showed a spectacular expansion in eukaryotes [4].

The superfamily is divided into five major families: Arf, Rab, Ran, Ras and Rho [5]. Rab proteins form the largest branch of the Ras superfamily (see for review: [6-13]). This family is much diversified and can be further divided into at least 14 groups/subfamilies [6]. Rab proteins are best known as key regulators of intracellular vesicular transport and membrane trafficking in exocytic and endocytic pathways. Each Rab protein has a distinct subcellular location and is responsible for a specific transport step. Rab proteins from diverse eukaryotes cluster in a phylogenetic tree into at least eight groups showing similar function, and/or subcellular localisation, and sequences [14]. This co-segregation of Rab GTPases according to common functions rather than to taxonomic relationships indicates a conserved mechanism of Rab interaction with regulators/effectors across evolution and a rapid divergence of these functional groups in the early evolution of eukaryotes. During radiation of eukaryotes numerous duplications led to diversification of Rab proteins in particular lineages.

Evolution and diversification of Rab proteins were studied in a global aspect [14] and in particular species [15-20] and also in selected taxonomic groups [21-23]. However, evolution of particular Rab groups was not yet analysed in detail in wide-ranging taxonomic studies. We focused in this study on the VII functional group (in the classification of Pereira-Leal and Seabra [14]) containing Rab7 and Rab9, two related subfamilies, showing common localisation to late endosomal compartment.

Rab7 proteins are localised in late endosomes, lysosomes and phagosomes. They regulate vesicular traffic from early to late endosomes and from late endosome to vacuole/lysosome [24-35]. Moreover, Rab7 proteins participate in the maturation and biogenesis of lysosomes [34,36]. They are also involved in fusion of late endosomes and lysosomes with primary phagosomes in specialized phagocytes [37]. They regulate the maturation and biogenesis of phagosomes both in unicellular eukaryotes and macrophages [36,38-42].

Recently, a novel human isoform of Rab7, named Rab7b was described (the former Rab7 isoform is often called Rab7a). Lysosome-localised Rab7b is involved in monocytic differentiation of human acute promyelocytic leukemia cells and possibly, also in regulation of monocyte functions [43]. Moreover, it negatively regulates proinflammatory and antipathogenic Toll-like receptor 4 signalling in macrophages [44]. Two isoforms of Rab7 were found in *Paramecium *[45] and in five fungi species [22], three isoforms in *Trichomonas vaginalis *[18], four in *Lotus japonicus *[46], eight in *Arabidopsis thaliana *[14,15], and nine in *Entamoeba histolytica *[19].

Rab9 proteins are found only in late endosomes. They are important for lysosomal enzyme delivery and are a key mediator of vesicular transport from late endosomes to the trans-Golgi network (TGN) [24,47-50]. Moreover, they are responsible for the maintenance of specific late endocytic compartments and endosome/lysosome localisation [51]. It has also been found that Rab9 GTPases are a key component for the replication of several viruses, including HIV1, Ebola, Marburg, and measles making Rab9 a potential target for inhibiting replication of some viruses [52,53]. A second human isoform of Rab9, named Rab9b was reported [54] and additionally a human Rab9 pseudogene was identified [55].

Interestingly, Rab9 proteins were not yet reported so far in unicellular eukaryotes, plants and fungi. On the other hand, Rab7 subfamily belongs to the ancestral set of Rab present in the ancestor of eukaryotes and all extant descendents [2,56]. Previous global phylogenies of Rab subfamilies showed close relationships of Rab7 proteins and Rab9 proteins [5,14]. However, evolution of these subfamilies was not studied in detail and any stage of evolution of Eukaryota in which the Rab9 subfamily diverged from Rab7 subfamily was not specified. The functional diversity and the presence of many isoforms of Rab7 and Rab9 make it interesting to study evolution, duplication events and phylogenetic relationships of these closely related proteins.

## Results and discussion

### Taxonomic distribution of Rab7 and Rab9 proteins

Thorough and detailed searches of public databases based on sequence annotation and similarity enabled us to gather as many as 210 non-redundant sequences representing Rab7 and Rab9 proteins. The search showed that Rab7 proteins are represented in all supergroups of Eukaryota: Excavata, Plantae, Chromalveolata, Amoebozoa and Opisthokonta (including Fungi and Metazoa = Animalia). However, Rab9 proteins are present only in representatives of multicellular animals (Metazoa) and *Monosiga brevicolis*, a member of choanoflagellates, the closest known relatives to metazoans [57,58]. Assuming the taxonomic distributions we can assume that Rab7 proteins arose before the radiation of eukaryotes and Rab9 proteins must have branched later among metazoans and their relatives. Interestingly, Rab7b isoforms are found only in representatives of amphibians, birds and mammals.

### Global phylogeny of Rab7 and Rab9 proteins

**Figure 1:**
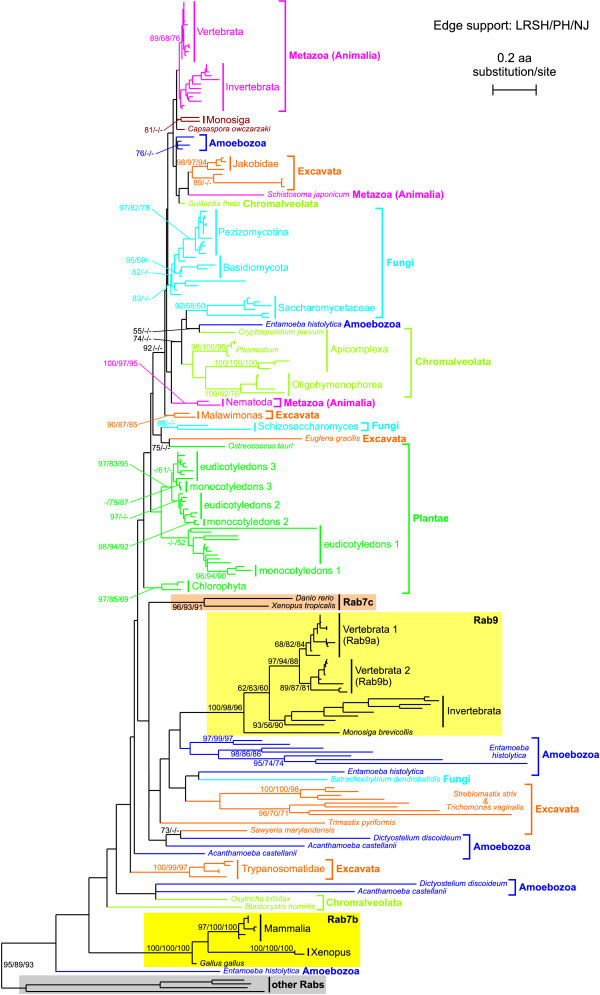
**The tree obtained in PHYML for Rab7 proteins and proteins classified in Rab9 and Rab7b subgroups (placed in the yellow rectangles)**. 'Rab7c' (in the orange rectangle), contains very divergent Rab7 proteins of vertebrates. 'Other Rabs' (in the grey rectangle), represent four human members of Rab23, Rab29, Rab32 and Rab38 subfamilies used as an out-group. Selected clades containing many members are shown in simplified way. Numbers at nodes, in the shown order, correspond to support values obtained for 1000 replicates in: the local rearrangement paired-sites method performed in TreeFinder (LRSH), the bootstrap analysis made in PHYML (PH) and bootstrap analysis based on neighbor joining method (NJ). Values of the bootstrap percentages lower or equal to 50% were omitted or indicated by a dash "-". Only selected support values, usually of deep branches, are shown. The expanded version of the tree is presented in Additional file 1.

To have a global view on phylogenetic relationships between all Rab7/Rab9 proteins we at first constructed a global ML tree based on their amino acid sequences. The tree is shown in Figure 1 (see Additional file 1 for the extended version). These sequences cluster together with a strong bootstrap support and clearly separate from representatives of the closest Rab subfamilies 23, 29, 32 and 38 [5,14]. In the tree we can distinguish clades that we expect should be monophyletic. Such monophyletic clades create many sequences of *Entamoeba histolytica *and sequences of Rab7b isoforms, Rab9 proteins, some subgroups of excavates (Trypanosomatidae, trichomonads, Jakobidae), subgroups of plants (Chlorophyta, angiosperms), most chromalveolates, Nematoda, most other metazoans, Saccharomycetaceae and most other fungi. A lot of these clades have a moderate or high bootstrap support or, for members of the same taxonomic group, are in a close neighbourhood in the tree. Interestingly, we named as Rab7c another well-supported clade grouping two highly diverged vertebrate sequences (*Danio rerio *and *Xenopus tropicalis*). In some slightly suboptimal trees we observed recovery of expected monophyly of some other clades that are not monophyletic in Figure 1. For instance, two separated sequences of chromalveolates (*Oxytricha trifallax *and *Blastocystis hominis*) were placed among other chromalveolates, three separated sequences of metazoans (*Schistosoma japonicum *and two nematods) were found in Metazoa clade and Rab7b from *Gallus gallus *grouped correctly with other amniotes.

However, the tree is generally poorly resolved, especially deep branches are very short and none of them has significant, if any, bootstrap support. Many single sequences, especially those of Excavata and Amoebozoa, are separated from other members of their own supergroup.

These relationships between Rab sequences that are inconsistent with species phylogeny are probably artificial and result from a heterogeneous rate of evolution of analysed sequences, insufficient phylogenetic information contained in these short sequences and the long-branch attraction artefact, LBA [59]. The LBA is especially evident in partition of the tree into two parts: one containing much diverged sequences and the other comprising less diverged sequences. However, these relationships are not supported by bootstrap analyses and should be considered uncertain. Inconsistencies of obtained phylogeny with species phylogeny are difficult to explain by a horizontal gene transfer or ancient gene duplications before radiation of eukaryotic supergroups and subsequent gene losses in the different lineages. There is no bootstrap support for relationships that would suggest these events. Moreover, the latter scenario requires many unparsimonious duplication and loss events, making this explanation highly unlikely. We performed phylogenetic analyses on seven alignment sets excluding gradually the most variable sites, but improvement or clarification could not be obtained because of the small number of sites necessary for a strong phylogenetic signal (data not shown).

To check consistency of the obtained Rab7/Rab9 gene phylogeny with the species phylogeny, we compared the topology of the found tree with the alternative topology assuming the most probable relationships between main groups of eukaryotes (see Additional file 2 and Additional file 3). These analyses revealed that when fast evolving sites are excluded from the data and more relatively conserved sites are present, the hypothesis of relationships between Rab proteins agreeable with species phylogeny can not be rejected or even is favoured.

Since Rab7 and Rab9 proteins show heterogeneous evolution rate and are susceptible to LBA we applied two programs proposed to such cases: PhyloBayes [60], Bayesian approach and PhyML-CAT [61], maximum likelihood approach. They use a mixture model describing across-site heterogeneities in the amino acid replacement patterns. It was shown that accounting for such site specific features should both improve a statistical fit [62] and alleviate phylogenetic artefacts due to long-branch attraction phenomena [63].

However, the application of these programs did not improve the obtained phylogenies (see Additional file 4 and Additional file 5). Although the monophyly of metazoan Rab7a proteins was recovered (i.e. three separated sequences of *Schistosoma japonicum *and two nematods were put into Metazoa clade), Rab7b from *Gallus gallus *adopted correct grouping with other amniotes and sequences of chlorophytes were clustered with other plants, the obtained trees are still poorly resolved with many unsupported branches. Moreover, other inconsistencies appeared, e.g.: choanoflagellate Rab7a is separated from metazoan sequences by trypanosomatid clade (the PhyloBayes tree), more chromalveolate sequences are separated from each other (the PhyML-CAT tree), sequence from Monosiga did not take basal position in Rab9 clade (both trees), and among plant clade unrelated sequences are located (both trees). Our analyses suggest that the phylogenetic signal in data is so weak and the sequences evolve with such a heterogeneous rate that even more sophisticated methods do not cope with inferring global phylogeny of analysed Rab proteins.

The obtained results show the presence of many Rab7 and Rab9 isoforms and duplicates in metazoans, plants and some unicellular eukaryotes. Therefore in subsequent sections we focused on relationships between Rab proteins belonging to these taxonomic groups.

### Phylogeny and duplications of metazoan Rab7 and Rab9 proteins

The group of Metazoa is the most abundant in different Rab7 and Rab9 isoforms and duplicates. The phylogeny based on amino acid sequences (Figure 1 and Additional file 1) revealed distinct and highly supported clades grouping metazoans sequences: Rab9 proteins (present in both invertebrates and vertebrates clustered significantly with *Monosiga brevicolis*), Rab7a (present also in invertebrates and vertebrates clustered with *Capsaspora *and *Monosiga*), Rab7b (present in Tetrapoda) and Rab7c proteins (present only in *Danio rerio *and *Xenopus tropicalis*). However, these clades are separated from each other. Very divergent sequences of Rab9, Rab7b and Rab7c proteins are also placed among very divergent sequences of excavates and amoebozoans, likely as a result of the LBA. Considering taxonomic distribution of Rab9 proteins we should expect that they diverged from Rab7 proteins before radiation of metazoans at the level of common ancestor of choanoflagellates and metazoan lineages. Similarly, the emergence of Rab7b isoforms from Rab7a proteins would precede the radiation of four-limbed vertebrates and Rab7c proteins would evolve from Rab7a at least before radiation of jawed vertebrates.

**Figure 2:**
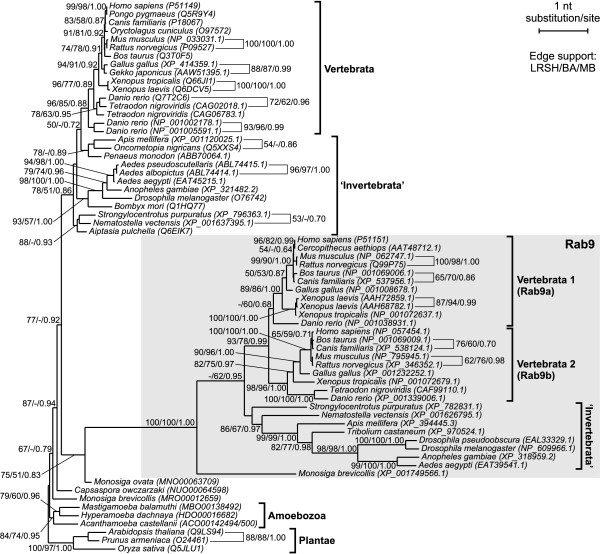
**The TreeFinder tree based on nucleotide sequences showing phylogenetic relationship of Rab9 subfamily (placed in the grey rectangle) with Rab7 proteins**. Numbers at nodes, in the shown order, correspond to support values obtained in TreeFinder by the local rearrangement paired-sites method (LRSH) and the maximum likelihood bootstrap analysis (BA), and posterior probabilities calculated in the MrBayes program (MB). Values of the bootstrap percentages and posterior probabilities lower or equal to 50% and 0.50, respectively, were omitted or indicated by a dash "-". Accession numbers for amino acid products were also included to be consistent with sequence names in the protein tree in Figure 1 and in Additional file 1.

**Figure 3:**
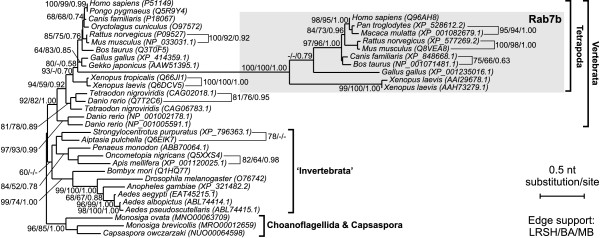
**The TreeFinder tree based on nucleotide sequences showing phylogenetic relationship of Rab7b subgroup (placed in the grey rectangle) with other Rab7 proteins**. Other explanations as in Figure 2.

**Figure 4:**
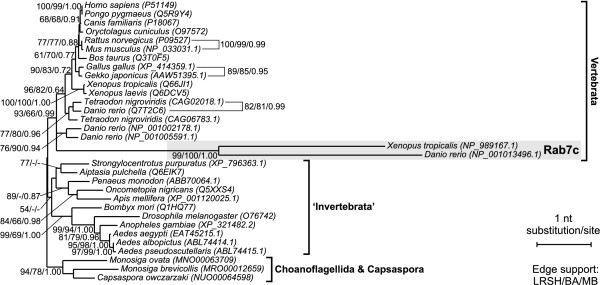
**The TreeFinder tree based on nucleotide sequences showing phylogenetic relationship of Rab7c subgroup (placed in the grey rectangle) with other Rab7 proteins**. Other explanations as in Figure 2.

In order to test the credibility of assumed relationships among metazoan Rab proteins we carried out additional phylogenetic analyses of their selected subgroups including potential sister sequences and appropriate out-groups (Figures 2, 3 and 4). These analyses were based on nucleotide sequences. They showed stronger phylogenetic signals than amino acid sequences and the trees obtained were better resolved than those constructed on the latter ones. Each subgroup was examined separately to avoid the LBA resulting from a high substitution rate of the analysed sequences. The trees obtained with ML and Bayesian approaches had almost identical topologies.

Since saturation of substitutions and base composition may violate reliability of results of phylogenetic analyses (especially based on nucleotide sequences) we carried out the appropriate tests to check it (see Material and Methods for details). However, the analysed whole nucleotide sets did not show significant saturation effect. We also did not observe a meaningful deviation in nucleotide composition between sequences in these sets.

The exclusive representation of Rab9 proteins by choanoflagellate *Monosiga brevicolis *and multicellular animals indicated that this subfamily branched from Rab7 near the divergence of choanoflagellate and metazoan lineages. In agreement with that, a very strongly supported Rab9 clade, shown on the tree in Figure 2, branched among choanoflagellate/metazoan clade of Rab7a proteins. Such a position is strongly supported by very high Bayesian posterior probabilities and moderate LRSH support at two external nodes comprising Rab9 and Rab7 sequences of Metazoa, *Monosiga ovata *and *Capsaspora owczarzaki*, a unicellular opisthokont closely related to animals and choanoflagellates [64,65]. Similarly, Rab7b proteins were identified only in the four-limbed vertebrates suggesting their divergence from Rab7a isoforms before radiation tetrapods. Actually, the clade of Rab7b proteins is placed among this group of vertebrates with moderate posterior probability support and very high LRSH support (Figure 3). The expected position of Rab7c GTPases represented only by sequences of *Danio rerio *and *Xenopus tropicalis *is also recovered (Figure 4). They branched at the base of vertebrate Rab7a clade with very high LRSH and posterior probability and moderate ML bootstrap support. The clade Rab7c has very high bootstrap values and contains none of representatives of amniotes and only the teleost fish and amphibian. Therefore a loss of this isoform can be assumed in the amniote lineage.

The phylogenetic reconstructions also revealed that subsequent duplications of Rab genes occurred in different lineages. Both the protein and gene trees include highly supported sister clades comprising Rab9a and Rab9b isoforms (Figure 2). Each of these clades groups Rab9 sequences of fishes, amphibians, birds and mammals suggesting that the duplication occurred among this subfamily prior to divergence of jawed vertebrates. Moreover, two clustered gene copies of both Rab9 and Rab7b are present in *Xenopus laevis *indicating additional duplications that occurred in this species (Figure 3). Since three Rab7a gene copies were found in *Danio rerio *and two in *Tetraodon nigroviridis*, it may suggest duplications in Rab7a isoforms among the teleost fish lineage.

To further resolve observed relationships between particular Rab genes we compared regions including these interesting Rab genes from key genomes of *Danio rerio*, *Tetraodon nigroviridis*, *Xenopus tropicalis*, *Gallus gallus *and *Canis familiaris *based on data available in Ensembl database [66]. The analyses showed that the Rab genes belonging to the same clade, i.e. Rab7a, Rab7b, Rab7c, Rab9a and Rab9b, were flanked by their own set of orthologous (syntenic) genes. It indicates that the genes clustered together in these clades have the common origin which is in agreement with high bootstrap values at their nodes in phylogenetic trees. It is worth to mention that the local conservation of orthologous gene content was also found for two Rab7c genes. Since they are highly diverged one could assume that they are grouped because of LBA and should be placed separately among the vertebrate Rab7a clade. However, the analysis of synteny allows assuming the common descent of these two genes.

No shared synteny with any compared genomes we found only for two genes from D. rerio encoding proteins with accession numbers NP_001005591.1 and NP_001002178.1. These genes are grouped together, take basal position in the tree and are separated from other Rab7a proteins. Probably they represent early duplicated Rab genes which were not retained in other vertebrates.

The observed duplications of Rab genes are probably a result of whole genome duplications (WGD) that occurred at least three times in the vertebrate lineage. The emergence of the Rab7c clade and the duplications among vertebrate Rab9 proteins mapped before splitting of jawed vertebrates could be related to one or two rounds (1R and 2R) of WGD (see e.g. [67] for a recent review and [68,69] for recent evidence). The third round of WGD (3R) occurred in teleost fishes [70-72], which is also reflected in fish Rab7a duplications. Observed Rab9 and Rab7b gene copies in *Xenopus laevis *are probably related to tetraploidization of its genome [73,74]. As a result of all these duplication events *Danio rerio, Xenopus laevis *and *Homo sapiens *possess as many as 6, 5 and 4 Rab7/Rab9 gene copies, respectively.

It is interesting that not all analysed taxa contain a full set of all Rab isoforms. For example, Rab7c clade is represented only by one fish (*Danio rerio*) and one amphibian (*Xenopus tropicalis*) sequence. Probably, different Rab gene copies were lost in different genera/species or these genes have not yet been identified. Such asymmetry in duplicated gene distribution is in agreement with results of Woods *et al*. [75] who found that many different duplicated genes were retained in *Danio *and *Tetraodon*, although similar numbers of duplicates remained in both genomes. Such differential retention of duplicate genes may have facilitated the isolation of nascent species formed during the vast radiation of teleosts and does not appear to be an exceptional phenomenon. Moreover, it seems even a desired one to accomplish speciation.

### Phylogeny and duplications of plant Rab7 proteins

**Figure 5:**
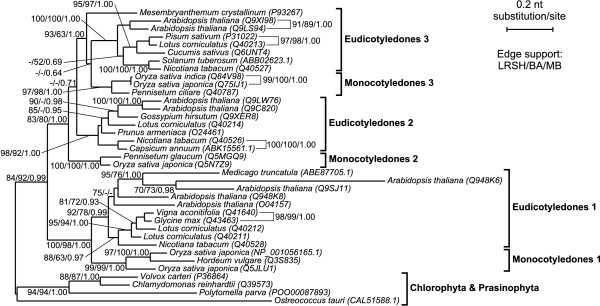
**The TreeFinder tree based on nucleotide sequences showing phylogenetic relationship among Rab7 proteins in plants**. Other explanations as in Figure 2.

Many Rab7 isoforms are also present in plant taxa. Both protein- and gene-based phylogenies revealed clear duplications of Rab7 genes that occurred in higher plants three times before divergence for monocots and eudicots giving three distinct Rab7 lineages (Figure 1 and Figure 5). The ML and Bayesian trees based on nucleotide sequence revealed almost identical topologies with many strongly supported clades. Apart from the early duplications of Rab7 in the evolution of higher plants, successive duplications must have happened among eudicots and monocots because additional copies are present in *Arabidopsis thaliana *(8 copies), *Lotus corniculatus *(4 copies) and *Oryza sativa *(4 copies).

The unravelled duplications events could be correlated with small-, large- or whole genome duplications recognized in angiosperms evolution. Analyses of *Arabidopsis thaliana *genome revealed three rounds of WGD in its lineage: before monocot-eudicot divergence (1R), after the divergence of monocots and eudicots (2R), and after the divergence of Brassicales and Malvales, but prior to the divergence of *Arabidopsis *and *Brassica *(3R) [76-81]. Early polyploidization before radiation of flowering plants was also found by Cui *et al*. [82]. The observed split of Rab7 gene lineages into three Rab7 lineages each containing monocots and eudicots genes could be reminiscent of these early duplications. Similarly, the clade comprising the *Medicago truncatula *sequence and four non-monophyletic *Arabidopsis thaliana *Rab7 sequences in the Rab7 lineage I may correspond to the duplication event (2R) before eurosid divergence [see also [83]]. Finally, particular *Arabidopsis thaliana *Rab7 proteins cluster together in pairs in all three lineages, thus indicating that the most recent WGD (i.e. 3R) occurred in the course of its genome evolution. The two separated Rab7 copies present in *Lotus corniculatus *in the lineage I were probably retained after WGD which occurred early in legume evolution [84]. The presence of two *Oryza sativa *Rab7 sequences in the lineage I significantly separated by the sequence of *Horedum vulgare *could be related with ancient WGD or other duplication events predating the divergence of the grass family [85-90].

### Duplications of Rab7 proteins in other eukaryotes

Several lineage-specific duplications events can also be deduced for some unicellular eukaryotes (Figure 1 and Additional file 1). Seven Rab7 copies are present in *Trichomonas vaginalis*. In this case all the copies form one unsupported clade branched to subsequent supported subclades. One of these includes the sequence of other excavate (*Streblomastix strix*) which could suggest that the duplication may have happened before radiation of Excavata for parabasalids and oxymonads. However, the identity of the sequence attributed to *Streblomastix strix *may be dubious since the EST library is highly contaminated with sequences from parabasalid species co-inhabiting the insect host together with oxymonad [91]. Therefore it is very likely that its close relationship to *Trichomonas vaginalis *sequences is best explained by a parabasalid rather than oxymonad provenance of this sequence.

Ten Rab7 proteins can be identified in *Entamoeba histolytica*. Most form one clade but only subclades show bootstrap support. Two Rab7 copies are present in *Schizosaccharomyces pombe *and three in each of *Paramecium *species (*P. tetraurelia *and *P. octaurelia*) analysed so far. (In phylogenetic tree in Figure 1 only two sequences from *Paramecium aurelia *are shown because its two sequences Q95UJ0 and ACJ09042.1 are identical at the amino acid level. These sequences are represented in the tree by the Q95UJ0 and are coded by different genes). However, duplicated sequences form a strongly supported clade only in the latter case. Two amoebozoans *Acanthamoeba castellani *and *Dictyostelium discoideum *have four and three Rab7 copies, respectively. In three cases the sequences of these two species are branched together, that suggests gene duplications before divergence of these lineages, although these clades do not have any bootstrap support. A very intriguing case is presented by two sequences of fungus *Batrachochytrium dendrobatidis *that are very distant in the tree. This separation may result from the LBA because one is very divergent.

Similarly to metazoans and plants, the presence of many Rab7 copies in some unicellular eukaryotes appeared as a result of WGD or other genome duplications that took place in a smaller scale. At least three successive whole-genome duplications occurred relatively recently in *Paramecium *lineage [92] and possibly one or more large-scale duplication events happened in *Trichomonas vaginalis *genome [93]. Relatively recent duplications were also reported for *Dictyostelium discoideum *genome [94] that seems to be especially susceptible to WGD [95].

### Evolutionary consequences of Rab duplications and their diversification

The presence of many Rab duplicates and isoforms raises a question about potential evolutionary consequences of Rab duplications. Duplicated sequences usually evolve significantly faster than unduplicated genes with a similar level of divergence, showing an early phase of relaxed constraints is in agreement with the view that gene duplications are a source of new protein functions [96,97]. Actually, we found that Rab9, Rab7b, Rab7c and some *Arabidopsis thaliana *(in the lineage 1) sequences showed elevated levels of substitutions that can be related with gaining of a new function or subfunctionalization [98].

Since Rab proteins are specialized to particular subcellular locations and functions, and several are tissue, organ- and developmental stage-specific, it was postulated that the diversification of Rab genes correlates with multicellularity of organisms [14]. The emergence of Rab9 before the radiation of Metazoa could fit this assumption. However, relationships between the Rab expansion and the multicellularity are not clear. The origin of Rab9 proteins responsible for vesicular transport and lysosomal enzyme sorting from late endosomes to the trans-Golgi network may have been an important step in improvement of digestion and degradation processes associated with phagocytosis, endocytosis, autophagy and apoptosis. Similarly to cell adhesion and signalling proteins that are otherwise restricted to metazoans, Rab9 was identified in one unicellular eukaryote,*Monosiga brevicolis*, the closest known relative to metazoans whose genome was completely sequenced [99]. It implicates that the common ancestor of metazoans and choanoflagellates already possessed several of the critical structural components used in modern Metazoa.

Additional duplication of Rab9 and Rab7 proteins that occurred in the early evolution of vertebrates and subsequently in teleost fishes and tetrapods can be connected to the functional diversification of these genes in vertebrates. The duplicated Rab might have acquired novel spatiotemporal roles related to expression in different cell types, to developmental stages or to environmental conditions. However, non-detailed functions or expression patterns for many Rab paralogous genes in given lineages or species were conclusively specified. Therefore, potential consequences of these Rab duplications in evolution of vertebrates are not clear. The specialization of function was found only for Rab7b isoforms identified in terrestrial vertebrates. Rab7a proteins usually regulate vesicular traffic from early to late endosomes and from late endosome to vacuole/lysosome while the Rab7b isoform regulates transport only to or from lysosomes. Moreover, it is selectively expressed in monocytes and probably is involved in their differentiation [43,44].

In order to estimate expected differences in expression profiles or levels of various Rab genes, we gathered data on expression profiles from UniGene database [100] and compared fractions of ESTs between different Rab isoforms or their duplicates. It should be noted that the expression data provide an approximate estimation of gene expression but are a good starting point for further investigations. Nevertheless, the data in UniGene expression profile of *Arabidopsis thaliana *genes lead to the same conclusions as data obtained in more exact experiments by Mazel A, *et al*. [101] as mentioned below (for the genes At.19280 and At.24625).

The comparisons clearly showed that Rab9a proteins always exhibit higher expression levels than Rab9b (Table S2 in Additional file 6). Similarly, Rab7a isoforms showed significant higher number of ESTs than Rab7b (Table S3 in Additional file 6). Since some species possess more than two Rab7 copies, we performed separate pair-wise comparison of their EST fraction (Table S4 in Additional file 6). Generally, these analyses also showed higher expression of Rab7a genes than other isoforms. Moreover, some variation of expression level was observed for different gene copies of the same Rab7 isoform (Table S4 in Additional file 6).

Similarly, the existence of many duplicated Rab7 isoforms in higher plants suggests their functional diversity. Interestingly, despite its small size, the *A. thaliana *genome (157 Mb) [102] retained as many as eight genes after duplication events suggesting a selective advantage of this variation.

Several experimental studies showed that duplicated Rab7 genes contributed to adaptation of plants to different environmental conditions and particular Rab7 copies acquired novel spatiotemporal roles and expression profiles. Borg *et al*. [46] found in *Lotus japonicus *that among four Rab7 genes, two of them (*rab7A *and *rab7B*) are preferably expressed in leaves, one (*rabt7C*) is most abundantly expressed in root nodules especially in middle stages of development and the fourth (*rab7D*) is constitutively expressed, representing a putative house-keeping gene. Mazel *et al*. [101] reported that one of the *Arabidopsis thaliana *Rab7 genes (*AtRabG3e*) showed higher expression in older roots and was induced during programmed cell death after treatment of intact leaves with superoxide and salicylic acid or infection with necrogenic pathogens. Transgenic plants that expressed this gene also showed increased tolerance to salt and osmotic stresses and a reduced accumulation of reactive oxygen species during salt stress. It was also found that expression of Rab7 gene both from *Oryza sativa *(*OsRab7*) and *Pennisetum glaucum *(*PgRab7*) was differentially regulated by various environmental stimuli such as cold, NaCl, dehydration and plant hormones [103,104]. Overexpression of *PgRab7 *gene enhanced tolerance to both ionic and osmotic stress in transgenic tobacco. It seems that at least some Rab7 isoforms are involved in developing tolerance towards salinity and dehydration in plants.

Our comparisons of plant EST fractions also showed differentiated expression profiles. We observed interesting relationships between the EST number of plant Rab7 proteins and the assignment of a given gene to a particular phylogenetic lineage (Table S5 in Additional file 6). Highest expression levels show genes clustered in the lineage 3, moderate levels show genes grouped in the lineage 2 and the lowest levels genes from the lineage 1. Moreover, we observed some organ-dependent expression patterns. The gene At.19280 has a significantly (p = 0.02) higher number of ESTs isolated from root than the gene At.24625 (13 ESTs vs. 1 EST) although these genes show indistinguishable global expression level (p = 0.38) and belong to the same phylogenetic lineage. Some organ-dependence expression pattern is indicated in this case. Actually, Mazel *et al*. [101] found the higher expression of the former gene in roots.

Less clear are expansions of Rab7 proteins in unicellular eukaryotes. Several explanations were proposed with reference to the whole Rab family [2,18,20]. Many Rab7 copies can be the result of large or whole genome duplication events and can be correlated with a highly complex endomembrane system or amoeboid lifestyle. Moreover, this phenomenon could be associated with the different possible substrates that are endocytosed by the parasite requiring a larger number of distinct cargo adaptors. On the other hand, the expansion of Rab7 proteins in single celled eukaryotes may be due to generation of diversity at the genome level instead of the transcriptome level since introns giving rise to different protein products by alternative splicing are rare in majority of these cells. These unique Rab GTPases may represent lineage-specific innovations similar to some other proteins involved in endocytosis [105].

Rab7 genes present in genomes of unicellular eukaryotes probably diversified into functionally and spatiotemporally different isoforms. Such a case was experimentally proven in *Entamoeba histolytica *[36,106,107] in which two Rab7 isotypes, EhRab7A and EhRab7B, showed distinct localisation and roles in biogenesis of lysosomes and phagosomes. EhRab7B is localised to late endosomes/lysosome and is involved in the formation and/or fusion to lysosomes, whereas EhRab7A is associated with the post-Golgi compartment containing cargos destined for lysosomes, and involved in fusion to late endosomes. Moreover, EhRab7 isotypes (EhRab7A-E) showed remarkable time- and stage-dependent recruitment to phagosomes during maturation indicating their sequential and coordinated influence on phagosome biogenesis.

Additionally, some other factors should be considered in the case of parasitic unicellular eukaryotes such as adaptive pressure that may increase the number of unique protein families including also that of Rab proteins. Trypanosomatid genomes encode approximately 20 Rab proteins including a group of three trypanosomatid unique Rabs and their functions have not been fully elucidated [17,108]. The authors point out to adaptive pressures placed upon these organisms to meet the demands of specialized host environments as well as a deep divergence due to early separation from model systems.

Since Rab proteins are important components of the endocytic network and are regulatory and signalling proteins interacting with many other proteins, the preservation of many copies is well suited to 'gene-balance' or a dosage constraint hypothesis. This hypothesis states that dosage-sensitive genes are preferentially retained after whole genome duplications rather than in cumulative small-scale gene duplications, especially if these genes cooperate in the same complex regulatory or interaction pathway and network [109-111]. In agreement with that Aury and coworkers [92] observed a clear co-retention of Rab GTP-ases with GTPase Activating Proteins (Supplementary Fig. S13 in [92]).

Our results indicate that new groups and paralogous of Rab7/Rab9 proteins emerged in the course of evolution of many lineages. Some Rab7 and Rab9 proteins acquired new experimentally proven specialized functions. The results also showed clear differences in expression between genes of particular subgroups of Rab7 and Rab9 proteins. However, additional experimental studies are required to determine detailed function for the particular Rab7 and Rab9 gene copies and to further assess the relationship between their expression and tissue, organ or development stage.

### Structural features of Rab7 and Rab9 sequences

**Figure 6:**
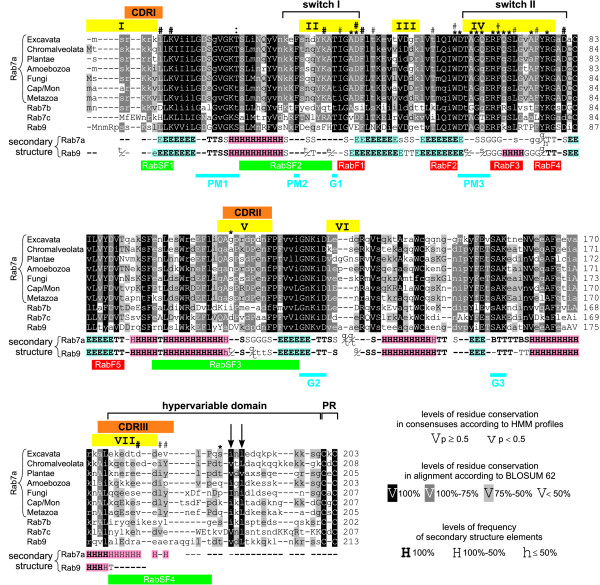
**Alignment of consensus sequences for Rab9 and different subgroups of Rab7 proteins**. Cap/Mon denotes Capsaspora/Monosiga. Highly conserved residues (with p ≥ 0.5) are shown in upper case and others are presented in lower case. Particular residues are shaded according to their levels of conservation in the alignment. Secondary structure consensuses for Rab7 and Rab9 proteins are shown below the alignment. Particular secondary structure elements are denoted by: H (α-helix), G (3_10_-helix), E (extended β-sheet), B (β-bridge), S (bend), T (turn), – (nonregular states and loops). The symbols of secondary structure elements are shown in different typefaces according to their frequency in the compared structures and α-helices and β-strands are marked with colored boxes. Different motifs, sequence elements and structural features related to Rab proteins are highlighted: G1 to G3 (conserved motifs involved in guanine nucleotide binding); PM1 to PM3 (conserved motifs responsible for binding and coordination Mg^2+ ^with phosphates groups); RabF1 to RabF5 (Rab family specific motifs); RabSF1 to RabSF4 (regions specific for particular subfamilies of Rab GTPases); switch I and II (flexible domains that substantially change their conformation upon exchange between GDP and GTP); hypervariable domain (HVD, unstructured region showing the highest level of sequence divergence among Rab proteins); PR (C-terminal prenylation motif); CDRI to CDRIII (complementary-determining regions); I to VII (seven regions showing substantial conformational variation among structures superimposed with Rab9 [53]). Some sites involved in interaction with regulators and effectors are pointed out above the alignment. Two key hydrophobic residues in the HVD region that make important contacts of Ypt1 with GDI [125] and Rab7 with REP1 [116] are marked with arrows. Other sites of Rab7 interacting with REP1 [116] are marked with *. Sites involved in the interaction of Rab7 with RILP are marked with # and **#** if their role was additionally confirmed by site-directed mutagenesis [119]. Another important site revealed by Harrison *et al*. [41] is marked with:.

In order to compare structural elements in sequences of different subgroups of Rab7 and Rab9 proteins we aligned consensus sequences generated from HMM profiles based on respective multiple sequence alignments (Figure 6). In this study we compared ten sets of the following proteins: Rab9, Rab7c, Rab7b and Rab7a dividing them into seven taxonomic subgroups. In Figure 6 we highlighted different conserved and unique sequence elements, motifs and sites that are responsible for interactions of Rab proteins with various regulators and effectors (see for review: [9,14,21,112,113]).

#### Ras- and Rab-conserved motifs

The most conserved regions, present not only in Rab proteins but also in other members of the Ras superfamily, contain six motifs: three (named G1 to G3) involved in guanine nucleotide binding and three (termed PM1 to PM3) responsible for binding and coordination magnesium ion with phosphates groups. These motifs are also conserved across the analysed sequences. Almost 85% of their sites are occupied by residues conserved in 100% across the alignment according to the BLOSUM62 matrix. Extensive comparative sequence analysis revealed also the presence of five Rab-conserved motifs (called RabF1 to RabF5), which may be used to designate this family [14,21]. In the analysed set, almost 60% of their sites are occupied by 100%-conserved residues. Some deviations from the full conservation observed in conserved motifs concern in almost all cases the substitutions that do not change the properties of the amino acid residues. The RabF1, -F3 and -F4 motifs are placed in two switch regions or in their neighbourhood. The regions are characteristic for small GTPases and correspond to flexible domains (loops) that substantially change their conformation upon exchange between GDP and GTP.

#### Regions specific for particular Rab subfamilies

Apart from the regions conserved for all Rab proteins, four other regions specific for particular subfamilies of Rab were identified [14,21,114]. They were named RabSF1 to RabSF4. These motifs show clear variation between Rab subfamilies and can be regarded as unique characteristics for each Rab subfamily. Both RabF and RabSF motifs are involved in simultaneous interaction with different regulators and effectors. It is postulated that they bind to RabF regions to discriminate between active/inactive nucleotide-bound conformations and simultaneously interact with RabSF regions for specificity. Determination of several structures of Rab proteins with their regulators or effectors revealed that in such interactions participate switch and interswitch regions, RabSF1, -SF3 motifs and CDRs (complementary-determining regions, overlapping RabSF1, -SF3 and -SF4 motifs) [115-119]. Moreover, all four RabSFs, RabF1 and -F4 motifs turned out to be important in correct subcellular targeting and functioning of many Rab GTPases in hybrid experiments exchanging these domains between different Rab proteins [120-124]. Therefore, we should expect potential differences between subgroups of Rab7/Rab9 proteins just in these above mentioned regions. Actually, in the analysed set, only 32% of RabSF1, RabSF2 and RabSF3 sites are occupied by 100%-conserved residues and none in the case of RabSF4. Moreover, the CDRs also show a very low level of conservation across the analysed alignment (Figure 6).

In Figure 6 we also marked seven regions, numbered from I to VII that show substantial conformational variation among structures of Rab proteins when superimposed with Rab9 [53]. Figure 6 includes also marked sites involved in interaction with regulators and effectors [41,116,119]. They usually fall into less conserved regions in the alignment. 100% conserved-sites constitute only 23% of the former regions and 38% of the sites involved in the interactions. Such variation suggests that these less conserved sites may be responsible for interaction with different regulators or effectors and differentiation of Rab functions.

#### C-terminal region

Rab proteins exhibit also a characteristic C-terminal prenylation motif that differs from the motifs found in Ras and Rho families, i.e. CAAX, where C denote cysteine, A – aliphatic residue and X – any residue [14]. The majority of Rab prenylation motifs, needed for the addition of geranylgeranyl groups and attachment of a protein to a membrane, occur in one of the exemplary combinations: XXCC, XCCX, CCXX, CCXXX or XCXC. Such two cysteines motifs were also found in all full Rab sequences analysed in this study. Interestingly, most amoebozoan Rab7a proteins, all Rab7b and all Rab9 sequences possessed motifs with two immediately adjacent cysteines while most sequences of other groups usually comprised motifs with Cs separated by one not conserved residue.

Immediately upstream of the prenylation site is located an unstructured hypervariable domain (HVD), that contains the RabSF4 motif and shows the highest level of sequence divergence among Rab proteins [122]. This region is also poorly conserved in analysed subgroups of Rab7/Rab9 proteins and is quite short (26 to 32 amino acid residues). It is assumed that shorter domains are bound more tightly to the surface of GDI (cytosol-resided GDP dissociation inhibitor responsible for sequestering a Rab protein in cytosol from membrane). The only conserved sites in the HVD region are occupied by two hydrophobic residues [112]. They make important contacts of Ypt1 with GDI [125] and Rab7 with REP1, an escort protein responsible for delivery of the GDP-Rab to an appropriate membrane [116]. Such two hydrophobic residues were found by us in the analysed sequences of Rab7/Rab9 proteins. Other interesting features of HVD is its high content of glycine and proline residues, that contribute to helix breakage and are responsible for generation of the extended structure important for GDI binding [125], prenylation of Rab GTPases [116] and probably other protein interactions. Interestingly, such residues are moderately present only in Rab7a proteins upstream of two hydrophobic residues and prenylation motifs but are absent from consensuses of Rab7b, Rab7c and Rab9 proteins.

The great variation of HVD suggests that this region is pivotal for Rab proteins localisation [122]. Actually, hybrid experiments replacing the region between different Rab proteins showed their incorrect targeting [120-123]. However, recent analyses revealed that the hypervariable region did not represent a general targeting domain and the Rab-effectors/regulators interaction is likely more complex and involves additional domains [124].

#### Sites discriminating different subgroups of Rab7 and Rab9 proteins

Detailed inspection of the alignment enabled us to identify individual sites containing non-conservative substitutions discriminating Rab7 and Rab9 subfamilies. For example, position 37 in the Rab9 consensus is occupied by aspartic acid whereas in Rab7 dominate hydroxylated residues. Moreover, hydrophobic and aromatic phenylalanine at position 41 of Rab9 consensus aligns with polar, mostly basic residues while valine at position 115 corresponds to prolines in other Rab proteins. Rab7b and Rab7c proteins contain at position 8 aspartic acid and histidine, negatively- and positively-charged residues, respectively, while other subgroups comprise uncharged, hydrophobic leucine. Furthermore, basic histidine at position 42 of Rab9 GTPases is aligned with small polar threonine of Rab7b consensus while tiny alanines are present in consensuses of other subgroups. Similarly, at position 77 of Rab9 consensus is basic arginine, whereas this position is occupied by hydrophobic valine in Rab7b proteins and by glycine in other subgroups. The residues discriminating Rab9 are placed in switch regions. In agreement, the greatest structural dissimilarity between Rab7 and Rab9 structures was just found in active switch conformations [126].

Wittmann and Rudolph [118] and Lal *et al*. [18] identified in CDRII/RabSF3 region four-residue insertion in Rab9 and Rab7 GTPases compared to other Rab proteins. The insertion renders the loop in this region more flexible [118]. However, the insertion can not be a unique feature of Rab7 and Rab9 proteins because we found it also in Rab29, Rab32 and Rab38 GTPases that are closely related to them. On the other hand, Rab7b proteins have a unique deletion (of one residue) at position 115 in comparison with other subgroups.

Based on the Rab records retrieved from the PDB database [127] we calculated consensuses of secondary structure for Rab7a and Rab9 proteins (Figure 6). The only significant difference concerns the presence of the short α-helix flanked by 3_10_-helices in the switch II region of Rab9. Due to such a structure a hydrophobic tetrad is formed resembling an effector-discriminating epitope [118]. This tetrad may contribute to differentiated recognition of effectors by these proteins.

The signal for the specific role and localisation of various Rab proteins is probably complex, involves many regions and could be achieved by relatively small differences in their structures. The identified non-conserved regions and structural dissimilarities of Rab7/Rab9 proteins may be a good starting point to further functional and structural studies.

## Conclusion

Rab7 proteins are widely distributed in almost all supergroups of Eukaryota and likely evolved before the radiation of eukaryotes. Rab9 proteins have more narrow taxonomic distribution and diverged from Rab7 GTPases before divergence of choanoflagellates and metazoans. The Rab proteins were additionally duplicated in vertebrates (Rab7 and Rab9) and in higher plants (Rab7). Interestingly, some representatives of excavates, ciliates and amoebozoans also comprise a substantial number of Rab7 copies. The emergence of the Rab9 subfamily and the subsequent duplication of genes encoding Rab7 and Rab9 may suggest their functional diversification and specialization. Actually, for some of these proteins such functions were already found and distinct expression levels were determined for different Rab genes. Apart from preserved conserved regions and motifs typical of Rab family, Rab7/Rab9 proteins have non-conserved sequences and structural features, that may be responsible for diversification of their functions and interactions with effectors and regulators. Rab7/Rab9 GTPases show concordant diversification at the phylogenetic, expression and sequence/structural levels. The obtained results are good starting point to further detail experimental studies which should fully determine functional specialization of these GTPases and relationship of their expression to tissue, organ, development stage or environmental response.

## Methods

### Collection of sequences and alignments construction

The analysed set of 210 sequences was obtained by thorough and detailed searches of public databases: GenBank [128], UniProt [129] and TBestDB [130] based on sequence annotation and similarity searches made by BLAST. Sequences annotated as Rab7 or Rab9 or sequences showing significant similarity to these proteins were included in the set. The membership of these sequences to the particular Rab subfamilies (Rab7: cd01862 or Rab9: cd04116) was verified based on Conserved Domain Database (CDD) searches [131]. Misannotated sequences were described in details in Additional file 7. Incomplete and redundant sequences were removed from the final set. We also included in the analyses four human sequences representing Rab23, Rab29, Rab32 and Rab38 subfamilies. These subfamilies show the closest relationship to Rab7 and Rab9 subfamilies among all Rab subfamilies [5,14] and therefore we chose them as an out-group in phylogenetic analyses. Accession numbers of all sequences used in the analyses are shown in Additional file 1.

All amino acid alignments were obtained in the MAFFT program using slow and accurate algorithm L-INS-i with 1000 cycles of iterative refinement [132]. Nucleotide sequences of selected subgroups of Rab proteins were aligned based on corresponding amino acid alignments. All resulting alignments were edited manually and corrected in GeneDoc [133] and the sites suitable for further phylogenetic analyses were extracted from the alignments with Gblocks 0.91b assuming less stringent criteria [134]. As a result, variable and poorly aligned sites, mainly in N- and C-terminal ends, were omitted from final alignments used in phylogenetic analyses. Gaps both in amino acid and nucleotide sets were treated as missing data by the applied phylogenetic programs.

In the preliminary studies we inferred trees from different data sets: the whole alignments (as they were in databases) and alignments with exclusion of all the sites containing at least one gap (complete-deletion approach). We also performed alignments with different number of excluded gaps. However, such data did not improve obtained phylogenies. Finally, we relied on more objective result of GBlocks and some manual corrections.

### Phylogenetic analyses

In phylogenetic analyses based on the set of 210 aligned amino acid sequences, we applied the JTT+I+Γ substitution model (seven rate categories) as proposed by the ProtTest program 1.4 [135] according to the Akaike Information Criterion (AIC), the second-order AIC and the Bayesian Information Criterion (BIC). In the case of the aligned nucleotide sequences of selected subgroups of Rab GTPases, we used separate models for each codon position: GTR+I+Γ or GTR+Γ as were suggested by Modeltest [136] according to the Akaike Information Criterion (AIC).

To find a tree close to optimal and avoid a trap of local optimum in global tree searches, the tree for 210 amino acid sequences was sought in several stages. At first, the maximum likelihood (ML) tree was constructed in PHYML [137] and the neighbour joining (NJ) tree was inferred in the neighbour program from the PHYLIP package 3.67 [138] based on the JTT+I+Γ(7) distance matrix calculated in Tree-Puzzle 5.2 [139]. Next, a set of 1000 start tree topologies was generated in TreeFinder [140] assuming the resulting ML and NJ tree as center trees. We generated 100 trees for each of five topological distances: 7, 10, 15, 20 and 25 NNI steps for each of these two center trees. We imposed topological constraints on the generated trees fixing such phylogenetic relationships that were supported by bootstrap values equal to or higher than 75% in a bootstrap tree. The bootstrap tree for the approach with the ML center tree was the consensus of 1000 ML trees calculated in PHYML whereas the bootstrap tree for the approach with the NJ center tree was the consensus of 1000 NJ trees calculated in the neighbour program based on JTT+I+Γ(7) distance matrices obtained in Tree-Puzzle. The 1000 generated trees were used as start ones for global tree search in PHYML (now with none constraints). We also conducted analyses with the NJ tree and the default BIONJ start tree in this program. The selected top 25 best topologies according to the maximum likelihood value were again used as start trees to PHYML, and subsequent iterations were carried out until the maximum likelihood value of resulting trees did not increase. The obtained best tree regarded as the final tree is presented in Figure 1 and in Additional file 1. Edge support of the tree was assessed by the aforementioned bootstrap analysis based on ML and NJ method assuming 1000 replicates and the local rearrangement paired-sites method (LRSH) with 1000 replicates made in TreeFinder.

Additionally, for amino acid alignments, we applied two programs: PhyloBayes [60], Bayesian approach and PhyML-CAT [61], maximum likelihood approach that use a mixture model describing across-site heterogeneities in the amino acid replacement patterns. In PhyloBayes analysis, two independent Markov chains were run for 600 000 cycles assuming the CAT+Γ model with number of components, weights and profiles inferred from the data and five discrete categories for gamma distributed rates. After getting a convergence, the last 50 000 trees from each chain were collected to compute posterior consensus in MrBayes [141] (see Additional file 4). For PhyML-CAT analysis, the tree was inferred with the CAT+I+Γ model assuming 30 profile mixture categories, five rate categories and SPR heuristic search algorithm. Edge support was assessed by the approximate likelihood ratio test (aLRT) based on χ^2 ^and Shimodaira-Hasegawa-like procedure [142]. The minimum of these two support values was shown at nodes in the tree (see Additional file 5).

Phylogenetic trees based on aligned nucleotide sequences of selected subgroups of Rab proteins were inferred in the TreeFinder [140] and MrBayes 3.1.2 programs [141] assuming separate models of substitutions GTR+I+Γ or GTR+Γ for three codon positions. Seven and five rate categories in the maximum likelihood (TreeFinder) and the Bayesian (MrBayes) approach were assumed, respectively. Edge support of the trees was assessed in TreeFinder by the bootstrap analysis and the local rearrangement paired-sites method (LRSH), each assuming 1000 replicates. In the Bayesian inference of phylogeny we applied two simultaneous independent runs starting from random trees using 5 Markov chains. Trees were sampled every 100 generations from 50 million generations. In the final analysis we selected trees from, depending on the analysed subgroup of Rab proteins, from the last 18 to 25 million generations that reached stationary phase with the average standard deviation of split frequencies much below the value 0.01. The temperature parameter (Temp) was properly adjusted to improve efficiency of analysis and get convergence.

The topology of the tree based on 210 amino acid sequences was compared with the alternative topology according to the approximately unbiased test (AU) and Kishino-Hasegawa tests (KH, WKH) carried out in the Consel v0.1i program [143] assuming ten million replicates. We also used two nonparametric paired-sites tests: sign and Wilcoxon matched pairs test implemented in Statistica software [144]. Site-wise log-likelihoods for the analysed trees were calculated in Tree-Puzzle under the JTT+I+Γ model and assumption of seven rate categories. In this analysis, we considered seven sets of sites that were created by successive elimination of sites with the highest substitution rate in the given set.

We assumed a full optimization of model parameters in all the above analyses with the exception of calculation of 1000 distance matrices performed in Tree-Puzzle based on bootstrapped alignments when the parameters estimated for the real alignment were applied.

### Testing saturation and compositional homogeneity of nucleotide sequences

To estimate level of saturation in analysed sequences we applied method developed by Xia *et al*. [145] implemented in DAMBE [146]. The testing showed that there is a little saturation in the whole alignment for each of four analysed nucleotide data sets.

Homogeneity of nucleotide composition of each sequence in a given set was analysed by χ^2 ^test in R package [147] with p-value computed by Monte Carlo simulation to give more reliable results. In each of four sets only a few sequences (from 3 to 7) showed deviation from other sequences. Moreover, the significance of these differences was not very high because the p-values were about 0.02 and 0.03. Additionally, these deviated sequences were usually scattered over the tree and do not tend to group together. However, if some of them were clustered in the gene trees, they also were grouped in protein trees. Therefore such grouping does not result from a deviated nucleotide composition but rather from the common origin. We also compared GC content between different well defined subclades of the gene trees by Kruskal-Wallis and post-hoc pairwise Wilcoxon test but we found only one weakly significant difference between Rab7b clade and Choanoflagellida & Capsaspora clade with p-value = 0.042.

### Consensus sequences and secondary structure

The HMMER 2.3.2 software [148] was used to generate consensus sequences for Rab9 and different subgroups of Rab7 proteins from HMM profiles that were calculated for respective multiple sequences alignments. The alignment of consensus sequences was prepared in GeneDoc with the aid of alignments of original sequences obtained in MAFFT (see Collection of sequences and alignments section).

Secondary structure consensuses for Rab7 and Rab9 proteins were calculated based on the annotated secondary structures for the structures retrieved from the PDB database [127], for Rab7 proteins: 1KY2:A, 1KY3:A, 1T91:A, 1T91:B, 1T91:C, 1T91:D, 1VG0:B, 1VG1:A, 1VG8:A, 1VG8:B, 1VG8:C, 1VG8:D, 1VG9:B, 1VG9:D, 1VG9:F, 1VG9:H, 1YHN:A and for Rab9 proteins: 1S8F:A, 1S8F:B, 1WMS:A, 1WMS:B, 1YZL:A, 2OCB:A.

### Comparison of expression level of Rab7 and Rab9 genes

Data on expression profiles coming from analysis of EST counts for particular Rab7 and Rab9 genes were downloaded from UniGene database [100]. Frequencies of Rab ESTs were compared by the test of proportions and the Benjamini-Hochberg multiple comparisons procedure for controlling false discovery rate was used. These statistical analyses were conducted in the R package [147].

## Authors' contributions

PM carried out all analyses, interpreted the results and wrote the manuscript. EW has been involved in conceiving the idea, collecting the data entries, drafting the manuscript, correcting and revising it critically and has given final approval of the version to be published. Both the authors read and approved the final manuscript.

## Supplementary Material

Additional file 1 **The full PHYML tree for Rab7 and Rab9 proteins**. The maximum likelihood tree obtained in PHYML under the JTT+I+G(7) model for 210 amino acid sequences of Rab7 and Rab9 proteins. Click here for file

Additional file 2 **Tests of tree topologies**. Result of statistical tests comparing topologies of the obtained Rab7/Rab9 gene phylogeny with the species phylogeny. These analyses showed that when fast evolving sites are excluded from the data, the hypothesis assuming relationships between Rab proteins agreeable with species phylogeny can not be rejected or even is favoured. Click here for file

Additional file 3 **The competitive topology to the obtained ML tree of Rab7 and Rab9 proteins**. The tree was used in topology tests as a competitive topology to the found maximum likelihood tree of Rab7 and Rab9 proteins presented in Figure 1 and Additional file 1. Click here for file

Additional file 4 **The PhyloBayes tree**. The Bayesian tree obtained in PhyloBayes under the CAT+Γ (5) model for 210 amino acid sequences of Rab7 and Rab9 proteins. Click here for file

Additional file 5 **The PhyML-CAT tree**. The maximum likelihood tree obtained in PhyML-CAT under the CAT+I+Γ (5) model assuming 30 profile mixture categories for 210 amino acid sequences of Rab7 and Rab9 proteins. Click here for file

Additional file 6 **Expression analyses**. Tables containing results of expression analyses of genes coding for Rab7 and Rab9 proteins. Click here for file

Additional file 7 **Sequence misannotation**. List and description of sequences wrongly annotated as Rab7 or Rab9 proteins. They actually belong to other Rab subfamilies. Click here for file
