# Supplementary material for: Phylogeny and evolution of Rab7 and Rab9 proteins
Source: BMC Evol Biol. 2009 May 14;9:101. doi: 10.1186/1471-2148-9-101 (PMC2693434; doi:10.1186/1471-2148-9-101)
Supplement: Additional file 1 — The full PHYML tree for Rab7 and Rab9 proteins. The maximum likelihood tree obtained in PHYML under the JTT+I+G(7) model for 210 amino acid sequences of Rab7 and Rab9 proteins. [file 1471-2148-9-101-S1.pdf]

The maximum likelihood tree obtained in PHYML under the JTT+I+Γ(7) model for 210 amino acid sequences of Rab7 proteins and proteins classified in Rab7b and Rab9 subfamily (placed in the yellow rectangles). 'Rab7c', in the orange rectangle, contains very divergent Rab7 proteins of vertebrates. 'Other Rabs', in the grey rectangle, represents four human members of Rab23, Rab29, Rab32 and Rab38 subfamilies used as an out-group. Numbers at nodes, in the shown order, correspond to support values obtained for 1000 replicates in: the local rearrangement paired-sites method performed in TreeFinder (LRSH), the bootstrap analysis made in PHYML (PH) and bootstrap analysis based on neighbor joining method (NJ). Values of the bootstrap percentages lower or equal to 50% were omitted or indicated by a dash “-“. Names at thick vertical lines denote sequences that were contracted in the competitive tree presented in Additional file 3. The contracted clades show a high bootstrap support or contain sequences belonging to well defined taxonomical groups.

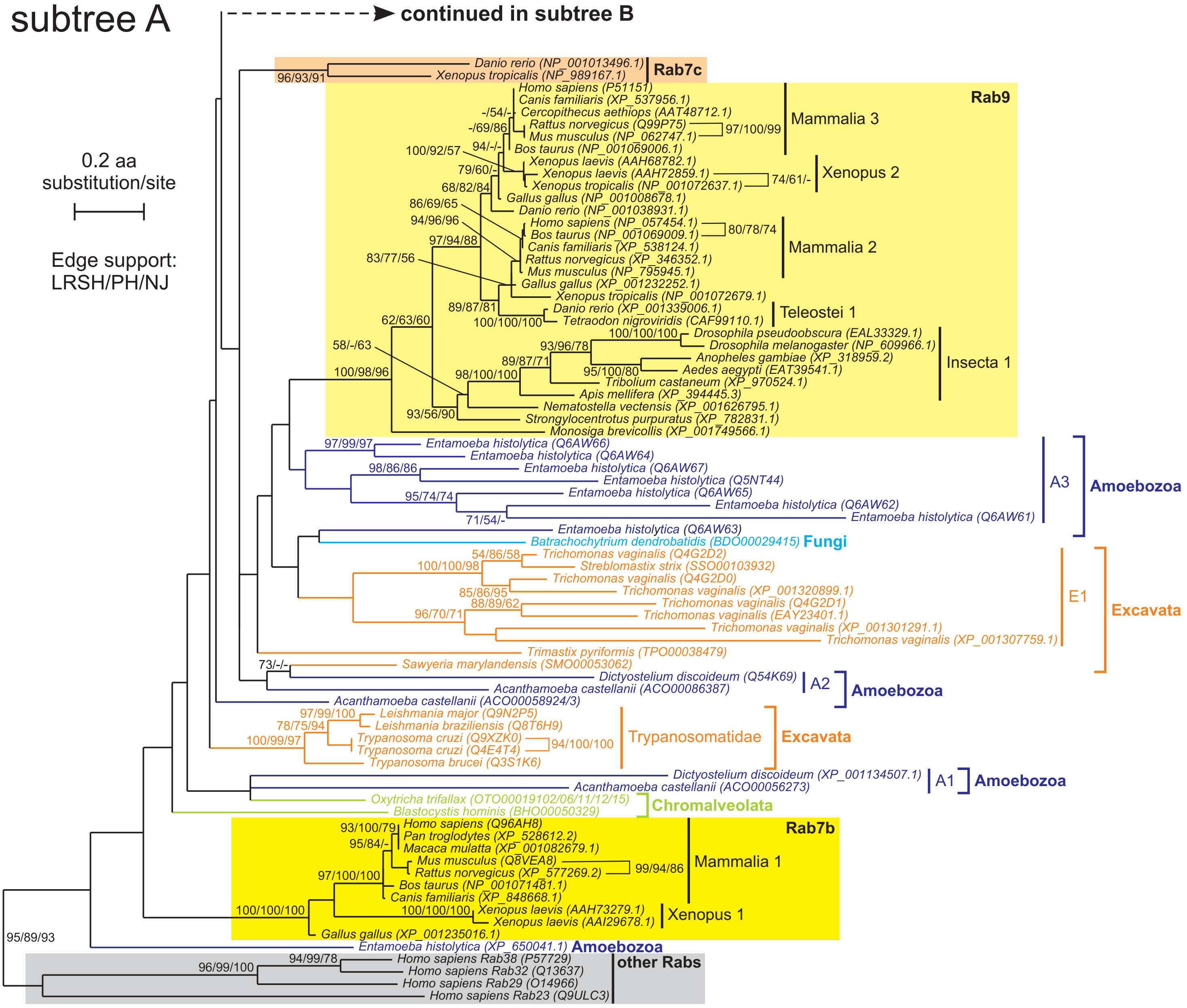
subtree A
continued in subtree B
0.2 aa substitution/site
Edge support: LRSH/PH/NJ
Rab7c
Rab9
Mammalia 3
Xenopus 2
Mammalia 2
Teleostei 1
Insecta 1
A3
Amoebozoa
Fungi
E1
Excavata
A2
Amoebozoa
Trypanosomatidae
Excavata
A1
Amoebozoa
Chromalveolata
Rab7b
Mammalia 1
Xenopus 1
Amoebozoa
other Rabs

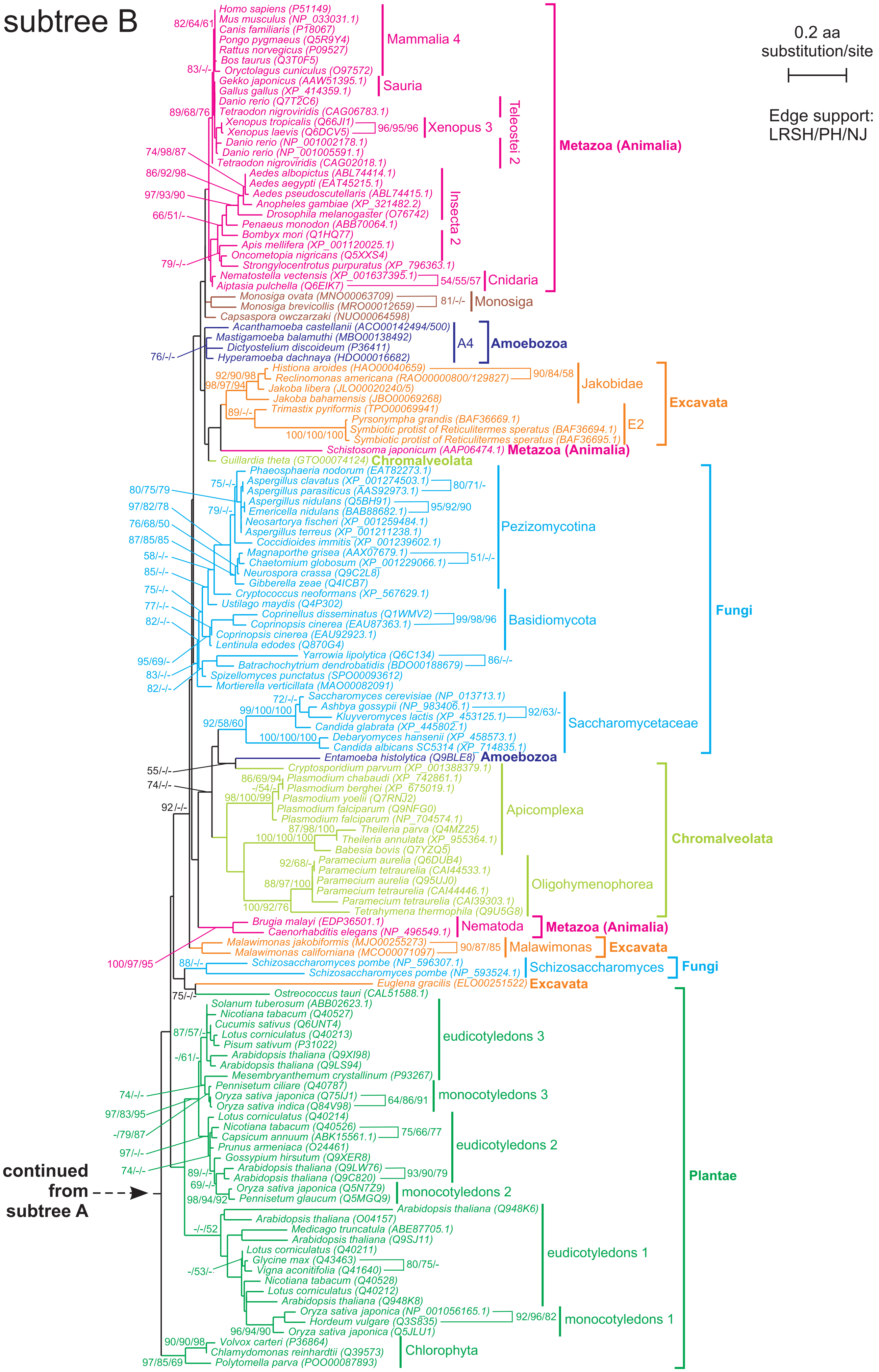

subtree B
0.2 aa substitution/site
Edge support: LRSH/PH/NJ
82/64/61
Homo sapiens (P51149)
Mus musculus (NP_033031.1)
Canis familiaris (P18067)
Pongo pygmaeus (Q5R9Y4)
Rattus norvegicus (P09527)
Bos taurus (Q3T0F5)
83/-/-
Oryctolagus cuniculus (O97572)
Mammalia 4
Gekko japonicus (AAW51395.1)
Gallus gallus (XP_414359.1)
Sauria
Danio rerio (Q7T2C6)
89/68/76
Tetraodon nigroviridis (CAG06783.1)
Xenopus tropicalis (Q66JI1)
Xenopus laevis (Q6DCV5)
96/95/96
Xenopus 3
Danio rerio (NP_001002178.1)
Danio rerio (NP_001005591.1)
Tetraodon nigroviridis (CAG02018.1)
Teleostei 2
74/98/87
86/92/98
Aedes albopictus (ABL74414.1)
Aedes aegypti (EAT45215.1)
97/93/90
Aedes pseudoscutellaris (ABL74415.1)
Anopheles gambiae (XP_321482.2)
66/51/-
Drosophila melanogaster (O76742)
Penaeus monodon (ABB70064.1)
Bombyx mori (Q1HQ77)
Apis mellifera (XP_001120025.1)
Oncometopia nigricans (Q5XXS4)
79/-/-
Strongylocentrotus purpuratus (XP_796363.1)
Insecta 2
Nematostella vectensis (XP_001637395.1)
Aiptasia pulchella (Q6EIK7)
54/55/57
Cnidaria
Metazoa (Animalia)
Monosiga ovata (MNO00063709)
Monosiga brevicollis (MRO00012659)
81/-/-
Monosiga
Capsaspora owczarzaki (NUO00064598)
Acanthamoeba castellanii (ACO00142494/500)
Mastigamoeba balamuthi (MBO00138492)
76/-/-
Dictyostelium discoideum (P36411)
Hyperamoeba dachnaya (HDO00016682)
A4
Amoebozoa
Histiona aroides (HAO00040659)
92/90/98
Reclinomonas americana (RAO00000800/129827)
90/84/58
98/97/94
Jakoba libera (JLO00020240/5)
Jakoba bahamensis (JBO00069268)
Jakobidae
89/-/-
Trimastix pyriformis (TPO00069941)
Pyrsonympha grandis (BAF36669.1)
100/100/100
Symbiotic protist of Reticulitermes speratus (BAF36694.1)
Symbiotic protist of Reticulitermes speratus (BAF36695.1)
E2
Excavata
Schistosoma japonicum (AAP06474.1)
Metazoa (Animalia)
Guillardia theta (GTO00074124)
Chromalveolata
80/75/79
75/-/-
Phaeosphaeria nodorum (EAT82273.1)
Aspergillus clavatus (XP_001274503.1)
Aspergillus parasiticus (AAS92973.1)
80/71/-
97/82/78
Aspergillus nidulans (Q5BH91)
79/-/-
Emericella nidulans (BAB88682.1)
95/92/90
76/68/50
Neosartorya fischeri (XP_001259484.1)
Aspergillus terreus (XP_001211238.1)
87/85/85
Coccidioides immitis (XP_001239602.1)
58/-/-
Magnaporthe grisea (AAX07679.1)
Chaetomium globosum (XP_001229066.1)
51/-/-
85/-/-
Neurospora crassa (Q9C2L8)
Gibberella zeae (Q4ICB7)
Pezizomycotina
75/-/-
Cryptococcus neoformans (XP_567629.1)
77/-/-
Ustilago maydis (Q4P302)
Coprinellus disseminatus (Q1WMV2)
82/-/-
Coprinopsis cinerea (EAU87363.1)
99/98/96
Coprinopsis cinerea (EAU92923.1)
Lentinula edodes (Q870G4)
Basidiomycota
95/69/-
Yarrowia lipolytica (Q6C134)
Batrachochytrium dendrobatidis (BDO00188679)
86/-/-
83/-/-
Spizellomyces punctatus (SPO00093612)
82/-/-
Mortierella verticillata (MAO00082091)
Fungi
72/-/-
Saccharomyces cerevisiae (NP_013713.1)
99/100/100
Ashbya gossypii (NP_983406.1)
Kluyveromyces lactis (XP_453125.1)
92/63/-
92/58/60
Candida glabrata (XP_445802.1)
100/100/100
Debaryomyces hansenii (XP_458573.1)
Candida albicans SC5314 (XP_714835.1)
Saccharomycetaceae
Entamoeba histolytica (Q9BLE8)
Amoebozoa
55/-/-
Cryptosporidium parvum (XP_001388379.1)
74/-/-
86/69/94
Plasmodium chabaudi (XP_742861.1)
-/54/-
Plasmodium berghei (XP_675019.1)
92/-/-
98/100/99
Plasmodium yoelii (Q7RNJ2)
Plasmodium falciparum (Q9NFG0)
Plasmodium falciparum (NP_704574.1)
87/98/100
Theileria parva (Q4MZ25)
100/100/100
Theileria annulata (XP_955364.1)
Babesia bovis (Q7YZQ5)
Apicomplexa
92/68/-
Paramecium aurelia (Q6DUB4)
Paramecium tetraurelia (CAI44533.1)
88/97/100
Paramecium aurelia (Q95UJ0)
Paramecium tetraurelia (CAI44446.1)
Paramecium tetraurelia (CAI39303.1)
100/92/76
Tetrahymena thermophila (Q9U5G8)
Oligohymenophorea
Chromalveolata
Brugia malayi (EDP36501.1)
Caenorhabditis elegans (NP_496549.1)
Nematoda
Metazoa (Animalia)
100/97/95
Malawimonas jakobiformis (MJO00255273)
Malawimonas californiana (MCO00071097)
90/87/85
Malawimonas
Excavata
88/-/-
Schizosaccharomyces pombe (NP_596307.1)
Schizosaccharomyces pombe (NP_593524.1)
Schizosaccharomyces
Fungi
Euglena gracilis (ELO00251522)
Excavata
75/-/-
Ostreococcus tauri (CAL51588.1)
Solanum tuberosum (ABB02623.1)
Nicotiana tabacum (Q40527)
Cucumis sativus (Q6UNT4)
87/57/-
Lotus corniculatus (Q40213)
Pisum sativum (P31022)
-/61/-
Arabidopsis thaliana (Q9XI98)
Arabidopsis thaliana (Q9LS94)
Mesembryanthemum crystallinum (P93267)
eudicotyledons 3
74/-/-
Pennisetum ciliare (Q40787)
Oryza sativa japonica (Q75IJ1)
97/83/95
Oryza sativa indica (Q84V98)
64/86/91
monocotyledons 3
-/79/87
Lotus corniculatus (Q40214)
Nicotiana tabacum (Q40526)
Capsicum annuum (ABK15561.1)
75/66/77
97/-/-
Prunus armeniaca (O24461)
Gossypium hirsutum (Q9XER8)
74/-/-
89/-/-
Arabidopsis thaliana (Q9LW76)
Arabidopsis thaliana (Q9C820)
93/90/79
eudicotyledons 2
69/-/-
Oryza sativa japonica (Q5N7Z9)
98/94/92
Pennisetum glaucum (Q5MGQ9)
monocotyledons 2
continued from subtree A
Arabidopsis thaliana (Q948K6)
Arabidopsis thaliana (O04157)
-/-/52
Medicago truncatula (ABE87705.1)
Arabidopsis thaliana (Q9SJ11)
Lotus corniculatus (Q40211)
-/53/-
Glycine max (Q43463)
Vigna aconitifolia (Q41640)
80/75/-
Nicotiana tabacum (Q40528)
Lotus corniculatus (Q40212)
Arabidopsis thaliana (Q948K8)
eudicotyledons 1
Oryza sativa japonica (NP_001056165.1)
Hordeum vulgare (Q3S835)
92/96/82
96/94/90
Oryza sativa japonica (Q5JLU1)
monocotyledons 1
90/90/98
Volvox carteri (P36864)
Chlamydomonas reinhardtii (Q39573)
97/85/69
Polytomella parva (POO00087893)
Chlorophyta
Plantae
