# Supplementary material for: Phylogeny and evolution of Rab7 and Rab9 proteins
Source: BMC Evol Biol. 2009 May 14;9:101. doi: 10.1186/1471-2148-9-101 (PMC2693434; doi:10.1186/1471-2148-9-101)
Supplement: Additional file 2 — Tests of tree topologies. Result of statistical tests comparing topologies of the obtained Rab7/Rab9 gene phylogeny with the species phylogeny. These analyses showed that when fast evolving sites are excluded from the data, the hypothesis assuming relationships between Rab proteins agreeable with species phylogeny can not be rejected or even is favoured. [file 1471-2148-9-101-S2.doc]

**Tests of tree topologies**

In order to check the expected phylogeny between analyzed sequences we compared topologies of the found tree (Figure 1 and Additional file 1) with the tree assuming presumable relationships of Rab9, Rab7b and Rab7c proteins to Rab7a GTPases. In the latter topology (see Additional file 3) we also included the most probable relationships between main groups of *Eukaryota* based on many references (see [1-7] and references therein). In the comparison of the topologies we considered seven sets of sites classified to different substitution rate categories (Table S1). The found topology was significantly better than the alternative one when fast evolving sites were included (rate categories 1-4, 1-5, 1-6 and 1-7). However, if the fast evolving sites were excluded and only sites of low rate categories 1-2 and 1-3 were considered, the alternative tree could not be significantly rejected by any test. Moreover, for the rate category 1-2, the log likelihood value of the alternative tree was even higher than the value of the best found tree. Finally, when the slowest evolving sites (category 1) were considered, the alternative topology had significantly higher its log likelihood value than the best found tree according to four applied tests. Interestingly, the percentage of sites favoring the alternative topology is quite high even for all the sites (i.e. 40%). This value rapidly increases to almost 78% when fast evolving sites are excluded from the alignment. These results show that the strong rejection signal comes from the fast evolving sites and when relatively more conserved sites are included, the hypothesis about relationships between Rab sequences proposed in Additional file 3 can not be rejected.

1. Baldauf SL, Roger AJ, Wenk-Siefert I, Doolittle WF: **A kingdom-level phylogeny of eukaryotes based on combined protein data.** Science 2000, **290**:972-977.
2. Baldauf SL: **The deep roots of eukaryotes.** Science 2003, **300**:1703-1706.
3. Hedges SB: **The origin and evolution of model organisms.** Nat Rev Genet 2002, **3**:838-849.
4. Cracraft J, Donoghue MJ (editors): *Assembling the Tree of Life.* Oxford University Press, New York; 2004
5. Keeling PJ, Burger G, Durnford DG, Lang BF, Lee RW, Pearlman RE, Roger AJ, Gray MW: **The tree of eukaryotes.** Trends Ecol Evol 2005, **20**:670-676.
6. Hackett JD, Yoon HS, Li S, Reyes-Prieto A, Rümmele SE, Bhattacharya D: **Phylogenomic analysis supports the monophyly of cryptophytes and haptophytes and the association of rhizaria with chromalveolates.** Mol Biol Evol. 2007, **24**:1702-1713.
7. Moreira D, von der Heyden S, Bass D, López-García P, Chao E, Cavalier-Smith T: Global eukaryote phylogeny: **Combined small- and large-subunit ribosomal DNA trees support monophyly of *Rhizaria*, *Retaria* and *Excavata*.** Mol Phylogenet Evol 2007, **44**:255-266.

Table S1. Comparison of the best found tree with the alternative topology for different site rate categories. Significant p-values less than 0.05 are in **bold**.

| site rate categories | number of sites | log likelihood value of the | | ΔlnL=  lnL_2_–lnL_1_ | % sites when lnL_2_>lnL_1_ | p-value of statistical tests | | | |
| --- | --- | --- | --- | --- | --- | --- | --- | --- | --- |
|  |  | best found tree (lnL_1_) | alternative tree (lnL_2_) |  |  | AU | KH/  wKH | sign | Wilcoxon |
| 1 | 18 | -243.3 | -241.0 | 2.3 | 77.8 | **0.027** | **0.013** | **0.034** | **0.031** |
| 1-2 | 41 | -1289.7 | -1285.2 | 4.5 | 56.1 | 0.112 | 0.153 | 0.532 | 0.664 |
| 1-3 | 76 | -3899.8 | -3970.8 | -71.0 | 44.7 | 0.070 | 0.056 | 0.422 | 0.119 |
| 1-4 | 94 | -5854.2 | -5963.7 | -109.5 | 42.6 | **4·10^-4^** | **0.018** | 0.180 | **0.025** |
| 1-5 | 116 | -9042.1 | -9250.0 | -207.9 | 39.7 | **4·10^-6^** | **0.001** | **0.033** | **6·10^-4^** |
| 1-6 | 143 | -14346.5 | -14571.8 | -225.3 | 40.6 | **6·10^-6^** | **0.001** | **0.030** | **7·10^-4^** |
| 1-7 | 161 | -19212.9 | -19455.5 | -242.6 | 40.4 | **1·10^-5^** | **0.001** | **0.018** | **4·10^-4^** |
