# Supplementary material for: Phylogeny and evolution of Rab7 and Rab9 proteins
Source: BMC Evol Biol. 2009 May 14;9:101. doi: 10.1186/1471-2148-9-101 (PMC2693434; doi:10.1186/1471-2148-9-101)
Supplement: Additional file 3 — The competitive topology to the obtained ML tree of Rab7 and Rab9 proteins. The tree was used in topology tests as a competitive topology to the found maximum likelihood tree of Rab7 and Rab9 proteins presented in Figure 1 and Additional file 1. [file 1471-2148-9-101-S3.pdf]

The tree used in topology tests as a competitive topology to the ML tree presented in Figure 1 and Additional file 1. The tree assumes parsimonious and probable relationships between analyzed sequences based on their taxonomical distribution and the recent views on phylogeny of eukaryotes (see Additional file 2 for references). The contracted clades are fully shown in Additional file 1.

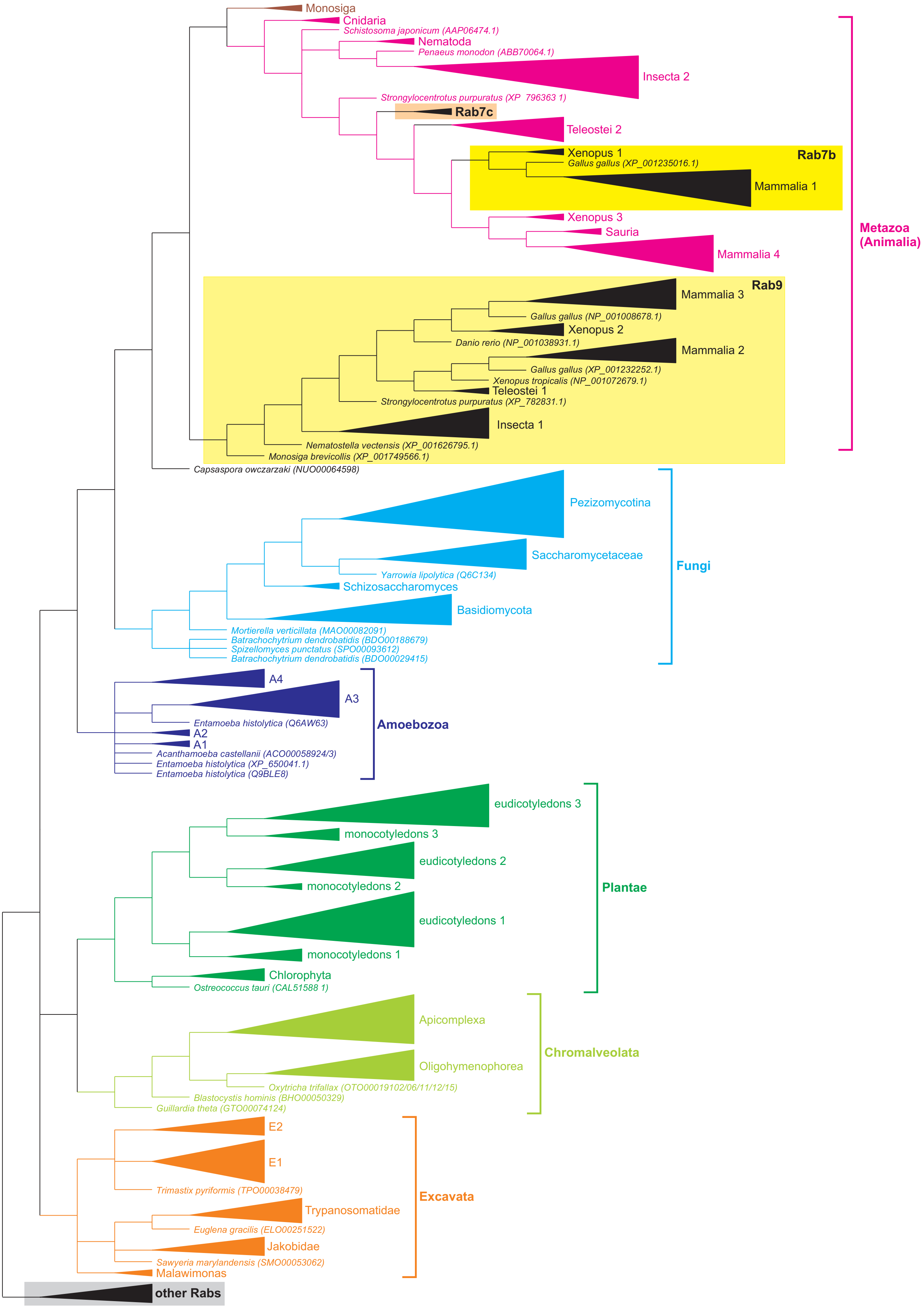

Monosiga
Cnidaria
Schistosoma japonicum (AAP06474.1)
Nematoda
Penaeus monodon (ABB70064.1)
Insecta 2
Strongylocentrotus purpuratus (XP 796363 1)
Rab7c
Teleostei 2
Xenopus 1
Gallus gallus (XP_001235016.1)
Rab7b
Mammalia 1
Xenopus 3
Sauria
Mammalia 4
Metazoa (Animalia)
Rab9
Mammalia 3
Gallus gallus (NP_001008678.1)
Xenopus 2
Danio rerio (NP_001038931.1)
Mammalia 2
Gallus gallus (XP_001232252.1)
Xenopus tropicalis (NP_001072679.1)
Teleostei 1
Strongylocentrotus purpuratus (XP_782831.1)
Insecta 1
Nematostella vectensis (XP_001626795.1)
Monosiga brevicollis (XP_001749566.1)
Capsaspora owczarzaki (NUO00064598)
Pezizomycotina
Saccharomycetaceae
Yarrowia lipolytica (Q6C134)
Schizosaccharomyces
Fungi
Basidiomycota
Mortierella verticillata (MAO00082091)
Batrachochytrium dendrobatidis (BDO00188679)
Spizellomyces punctatus (SPO00093612)
Batrachochytrium dendrobatidis (BDO00029415)
A4
A3
Entamoeba histolytica (Q6AW63)
Amoebozoa
A2
A1
Acanthamoeba castellanii (ACO00058924/3)
Entamoeba histolytica (XP_650041.1)
Entamoeba histolytica (Q9BLE8)
eudicotyledons 3
monocotyledons 3
eudicotyledons 2
monocotyledons 2
Plantae
eudicotyledons 1
monocotyledons 1
Chlorophyta
Ostreococcus tauri (CAL51588 1)
Apicomplexa
Chromalveolata
Oligohymenophorea
Oxytricha trifallax (OTO00019102/06/11/12/15)
Blastocystis hominis (BHO00050329)
Guillardia theta (GTO00074124)
E2
E1
Trimastix pyriformis (TPO00038479)
Excavata
Trypanosomatidae
Euglena gracilis (ELO00251522)
Jakobidae
Sawyeria marylandensis (SMO00053062)
Malawimonas
other Rabs
