# Supplementary material for: Phylogeny and evolution of Rab7 and Rab9 proteins
Source: BMC Evol Biol. 2009 May 14;9:101. doi: 10.1186/1471-2148-9-101 (PMC2693434; doi:10.1186/1471-2148-9-101)
Supplement: Additional file 4 — The PhyloBayes tree. The Bayesian tree obtained in PhyloBayes under the CAT+Γ (5) model for 210 amino acid sequences of Rab7 and Rab9 proteins. [file 1471-2148-9-101-S4.pdf]

The Bayesian tree obtained in PhyloBayes under the CAT+Γ(5) model for 210 amino acid sequences of Rab7 and Rab9 proteins. Numbers at nodes correspond to posterior probabilities. Values lower or equal to 0.50 were omitted.

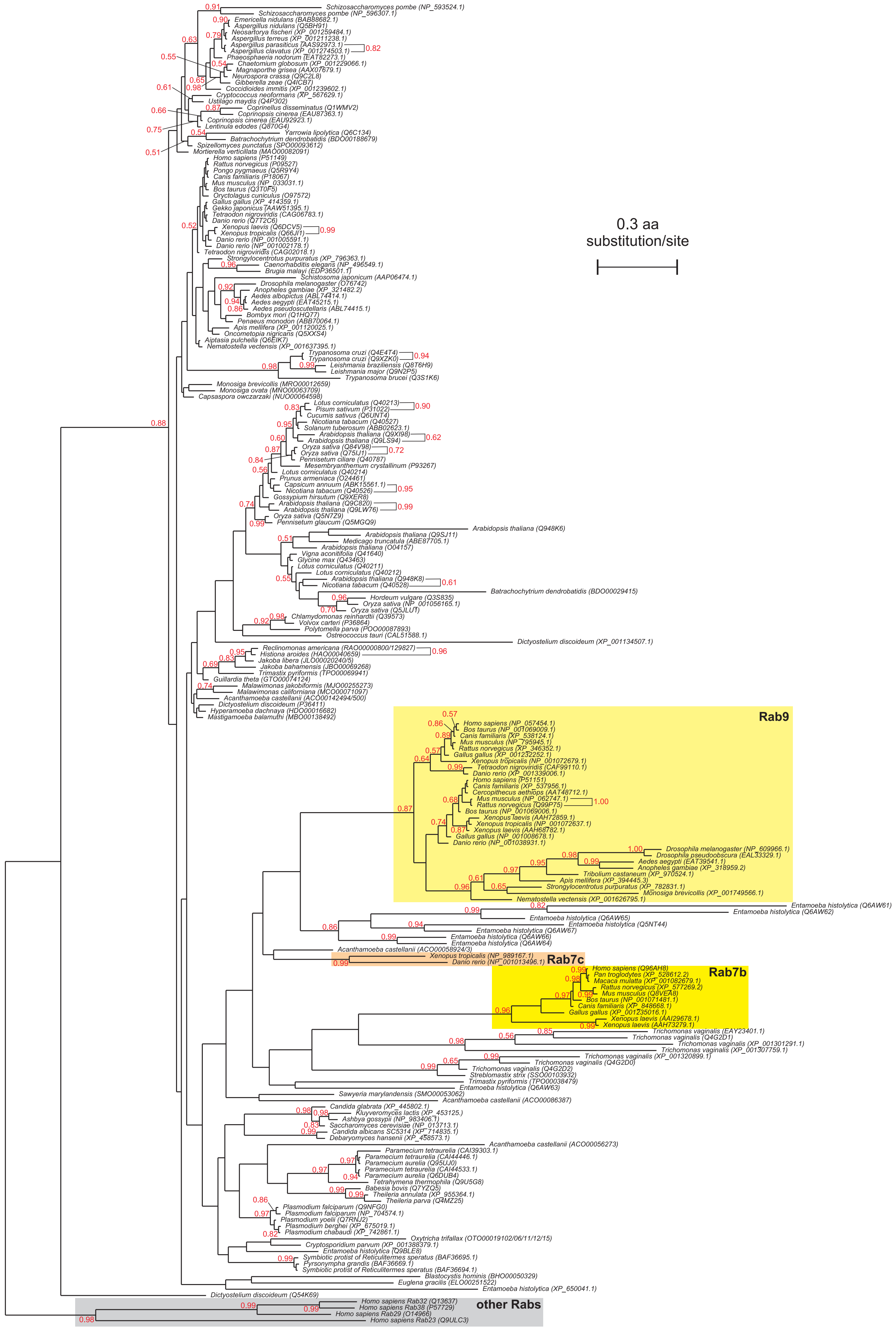

0.3 aa
substitution/site
Schizosaccharomyces pombe (NP_593524.1)
Schizosaccharomyces pombe (NP_596307.1)
Emericella nidulans (BAB88682.1)
Aspergillus nidulans (Q5BH91)
Neosartorya fischeri (XP_001259484.1)
Aspergillus terreus (XP_001211238.1)
Aspergillus parasiticus (AAS92973.1)
Aspergillus clavatus (XP_001274503.1)
Phaeosphaeria nodorum (EAT82273.1)
Chaetomium globosum (XP_001229066.1)
Magnaporthe grisea (AAX07679.1)
Neurospora crassa (Q9C2L8)
Gibberella zeae (Q4ICB7)
Coccidioides immitis (XP_001239602.1)
Cryptococcus neoformans (XP_567629.1)
Ustilago maydis (Q4P302)
Coprinellus disseminatus (Q1WMV2)
Coprinopsis cinerea (EAU87363.1)
Coprinopsis cinerea (EAU92923.1)
Lentinula edodes (Q870G4)
Yarrowia lipolytica (Q6C134)
Batrachochytrium dendrobatidis (BDO00188679)
Spizellomyces punctatus (SPO00093612)
Mortierella verticillata (MAO00082091)
Homo sapiens (P51149)
Rattus norvegicus (P09527)
Pongo pygmaeus (Q5R9Y4)
Canis familiaris (P18067)
Mus musculus (NP_033031.1)
Bos taurus (Q3T0F5)
Oryctolagus cuniculus (O97572)
Gallus gallus (XP_414359.1)
Gekko japonicus (AAW51395.1)
Tetraodon nigroviridis (CAG06783.1)
Danio rerio (Q7T2C6)
Xenopus laevis (Q6DCV5)
Xenopus tropicalis (Q66JI1)
Danio rerio (NP_001005591.1)
Danio rerio (NP_001002178.1)
Tetraodon nigroviridis (CAG02018.1)
Strongylocentrotus purpuratus (XP_796363.1)
Caenorhabditis elegans (NP_496549.1)
Brugia malayi (EDP36501.1)
Schistosoma japonicum (AAP06474.1)
Drosophila melanogaster (O76742)
Anopheles gambiae (XP_321482.2)
Aedes albopictus (ABL74414.1)
Aedes aegypti (EAT45215.1)
Aedes pseudoscutellaris (ABL74415.1)
Bombyx mori (Q1HQ77)
Penaeus monodon (ABB70064.1)
Apis mellifera (XP_001120025.1)
Oncometopia nigricans (Q5XXS4)
Aiptasia pulchella (Q6EIK7)
Nematostella vectensis (XP_001637395.1)
Trypanosoma cruzi (Q4E4T4)
Trypanosoma cruzi (Q9XZK0)
Leishmania braziliensis (Q8T6H9)
Leishmania major (Q9N2P5)
Trypanosoma brucei (Q3S1K6)
Monosiga brevicollis (MRO00012659)
Monosiga ovata (MNO00063709)
Capsaspora owczarzaki (NUO00064598)
Lotus corniculatus (Q40213)
Pisum sativum (P31022)
Cucumis sativus (Q6UNT4)
Nicotiana tabacum (Q40527)
Solanum tuberosum (ABB02623.1)
Arabidopsis thaliana (Q9XI98)
Arabidopsis thaliana (Q9LS94)
Oryza sativa (Q84V98)
Oryza sativa (Q75IJ1)
Pennisetum ciliare (Q40787)
Mesembryanthemum crystallinum (P93267)
Lotus corniculatus (Q40214)
Prunus armeniaca (O24461)
Capsicum annuum (ABK15561.1)
Nicotiana tabacum (Q40526)
Gossypium hirsutum (Q9XER8)
Arabidopsis thaliana (Q9C820)
Arabidopsis thaliana (Q9LW76)
Oryza sativa (Q5N7Z9)
Pennisetum glaucum (Q5MGQ9)
Arabidopsis thaliana (Q948K6)
Arabidopsis thaliana (Q9SJ11)
Medicago truncatula (ABE87705.1)
Arabidopsis thaliana (O04157)
Vigna aconitifolia (Q41640)
Glycine max (Q43463)
Lotus corniculatus (Q40211)
Lotus corniculatus (Q40212)
Arabidopsis thaliana (Q948K8)
Nicotiana tabacum (Q40528)
Batrachochytrium dendrobatidis (BDO00029415)
Hordeum vulgare (Q3S835)
Oryza sativa (NP_001056165.1)
Oryza sativa (Q5JLU1)
Chlamydomonas reinhardtii (Q39573)
Volvox carteri (P36864)
Polytomella parva (POO00087893)
Ostreococcus tauri (CAL51588.1)
Dictyostelium discoideum (XP_001134507.1)
Reclinomonas americana (RAO00000800/129827)
Histiona aroides (HAO00040659)
Jakoba libera (JLO00020240/5)
Jakoba bahamensis (JBO00069268)
Trimastix pyriformis (TPO00069941)
Guillardia theta (GTO00074124)
Malawimonas jakobiformis (MJO00255273)
Malawimonas californiana (MCO00071097)
Acanthamoeba castellanii (ACO00142494/500)
Dictyostelium discoideum (P36411)
Hyperamoeba dachnaya (HDO00016682)
Mastigamoeba balamuthi (MBO00138492)
Rab9
Homo sapiens (NP_057454.1)
Bos taurus (NP_001069009.1)
Canis familiaris (XP_538124.1)
Mus musculus (NP_795945.1)
Rattus norvegicus (XP_346352.1)
Gallus gallus (XP_001232252.1)
Xenopus tropicalis (NP_001072679.1)
Tetraodon nigroviridis (CAF99110.1)
Danio rerio (XP_001339006.1)
Homo sapiens (P51151)
Canis familiaris (XP_537956.1)
Cercopithecus aethiops (AAT48712.1)
Mus musculus (NP_062747.1)
Rattus norvegicus (Q99P75)
Bos taurus (NP_001069006.1)
Xenopus laevis (AAH72859.1)
Xenopus tropicalis (NP_001072637.1)
Xenopus laevis (AAH68782.1)
Gallus gallus (NP_001008678.1)
Danio rerio (NP_001038931.1)
Drosophila melanogaster (NP_609966.1)
Drosophila pseudoobscura (EAL33329.1)
Aedes aegypti (EAT39541.1)
Anopheles gambiae (XP_318959.2)
Tribolium castaneum (XP_970524.1)
Apis mellifera (XP_394445.3)
Strongylocentrotus purpuratus (XP_782831.1)
Monosiga brevicollis (XP_001749566.1)
Nematostella vectensis (XP_001626795.1)
Entamoeba histolytica (Q6AW61)
Entamoeba histolytica (Q6AW62)
Entamoeba histolytica (Q6AW65)
Entamoeba histolytica (Q5NT44)
Entamoeba histolytica (Q6AW67)
Entamoeba histolytica (Q6AW66)
Entamoeba histolytica (Q6AW64)
Acanthamoeba castellanii (ACO00058924/3)
Xenopus tropicalis (NP_989167.1)
Danio rerio (NP_001013496.1)
Rab7c
Rab7b
Homo sapiens (Q96AH8)
Pan troglodytes (XP_528612.2)
Macaca mulatta (XP_001082679.1)
Rattus norvegicus (XP_577269.2)
Mus musculus (Q8VEA8)
Bos taurus (NP_001071481.1)
Canis familiaris (XP_848668.1)
Gallus gallus (XP_001235016.1)
Xenopus laevis (AAI29678.1)
Xenopus laevis (AAH73279.1)
Trichomonas vaginalis (EAY23401.1)
Trichomonas vaginalis (Q4G2D1)
Trichomonas vaginalis (XP_001301291.1)
Trichomonas vaginalis (XP_001307759.1)
Trichomonas vaginalis (XP_001320899.1)
Trichomonas vaginalis (Q4G2D0)
Trichomonas vaginalis (Q4G2D2)
Streblomastix strix (SSO00103932)
Trimastix pyriformis (TPO00038479)
Entamoeba histolytica (Q6AW63)
Sawyeria marylandensis (SMO00053062)
Acanthamoeba castellanii (ACO00086387)
Candida glabrata (XP_445802.1)
Kluyveromyces lactis (XP_453125.)
Ashbya gossypii (NP_983406.1)
Saccharomyces cerevisiae (NP_013713.1)
Candida albicans SC5314 (XP_714835.1)
Debaryomyces hansenii (XP_458573.1)
Acanthamoeba castellanii (ACO00056273)
Paramecium tetraurelia (CAI39303.1)
Paramecium tetraurelia (CAI44446.1)
Paramecium aurelia (Q95UJ0)
Paramecium tetraurelia (CAI44533.1)
Paramecium aurelia (Q6DUB4)
Tetrahymena thermophila (Q9U5G8)
Babesia bovis (Q7YZQ5)
Theileria annulata (XP_955364.1)
Theileria parva (Q4MZ25)
Plasmodium falciparum (Q9NFG0)
Plasmodium falciparum (NP_704574.1)
Plasmodium yoelii (Q7RNJ2)
Plasmodium berghei (XP_675019.1)
Plasmodium chabaudi (XP_742861.1)
Oxytricha trifallax (OTO00019102/06/11/12/15)
Cryptosporidium parvum (XP_001388379.1)
Entamoeba histolytica (Q9BLE8)
Symbiotic protist of Reticulitermes speratus (BAF36695.1)
Pyrsonympha grandis (BAF36669.1)
Symbiotic protist of Reticulitermes speratus (BAF36694.1)
Blastocystis hominis (BHO00050329)
Euglena gracilis (ELO00251522)
Entamoeba histolytica (XP_650041.1)
Dictyostelium discoideum (Q54K69)
other Rabs
Homo sapiens Rab32 (Q13637)
Homo sapiens Rab38 (P57729)
Homo sapiens Rab29 (O14966)
Homo sapiens Rab23 (Q9ULC3)
