# Supplementary material for: Phylogeny and evolution of Rab7 and Rab9 proteins
Source: BMC Evol Biol. 2009 May 14;9:101. doi: 10.1186/1471-2148-9-101 (PMC2693434; doi:10.1186/1471-2148-9-101)
Supplement: Additional file 5 — The PhyML-CAT tree. The maximum likelihood tree obtained in PhyML-CAT under the CAT+I+Γ (5) model assuming 30 profile mixture categories for 210 amino acid sequences of Rab7 and Rab9 proteins. [file 1471-2148-9-101-S5.pdf]

The maximum likelihood tree obtained in PhyML-CAT under the CAT+I+Γ(5) model assuming 30 profile mixture categories for 210 amino acid sequences of Rab7 and Rab9 proteins. Different thickness of branches corresponds to given support value (p) that is the minimum of two support values assessed by the approximate likelihood ratio test (aLRT) based on χ2 and Shimodaira-Hasegawa-like procedure.

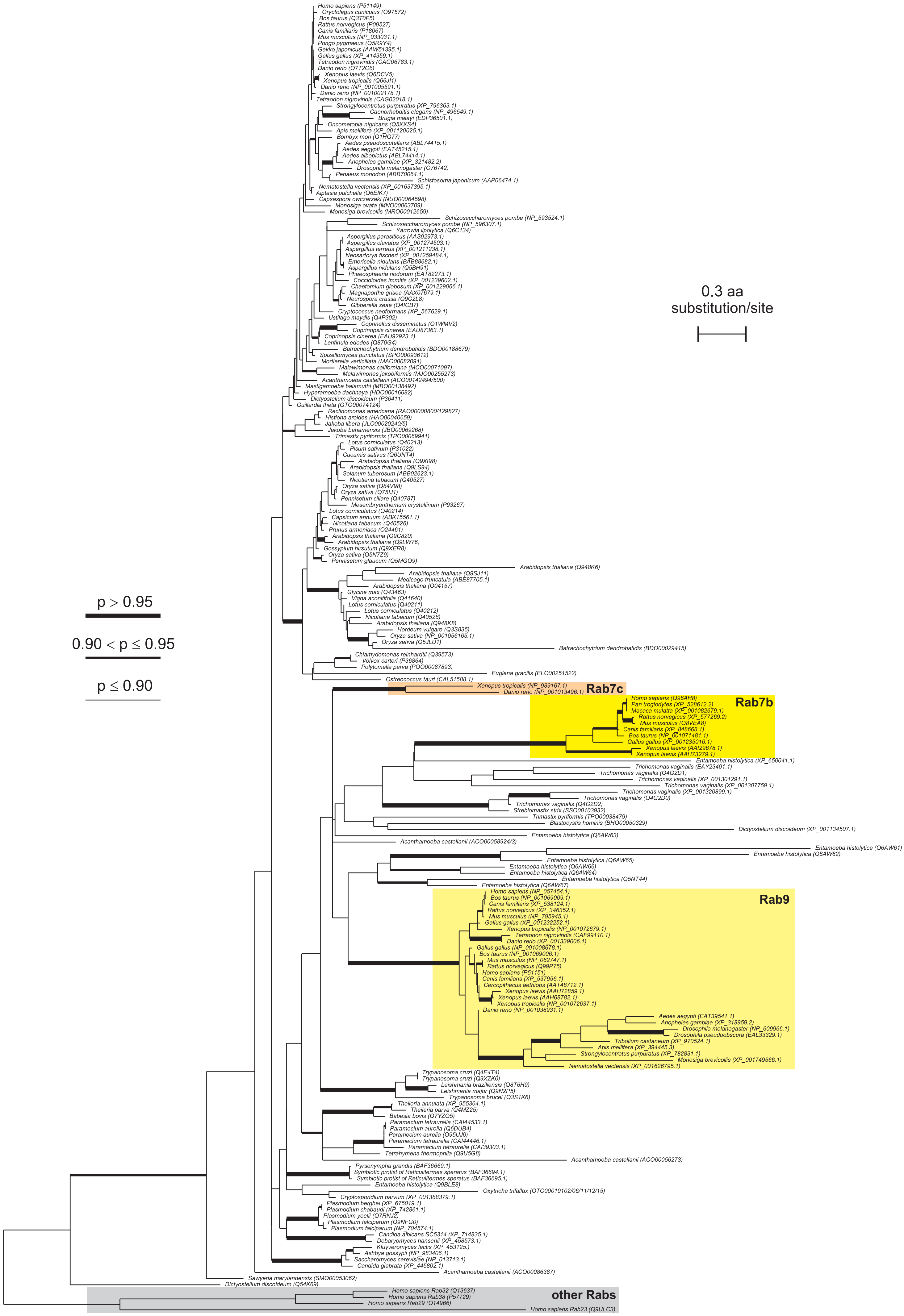
Homo sapiens (P51149)
Oryctolagus cuniculus (O97572)
Bos taurus (Q3T0F5)
Rattus norvegicus (P09527)
Canis familiaris (P18067)
Mus musculus (NP_033031.1)
Pongo pygmaeus (Q5R9Y4)
Gekko japonicus (AAW51395.1)
Gallus gallus (XP_414359.1)
Tetraodon nigroviridis (CAG06783.1)
Danio rerio (Q7T2C6)
Xenopus laevis (Q6DCV5)
Xenopus tropicalis (Q66JI1)
Danio rerio (NP_001005591.1)
Danio rerio (NP_001002178.1)
Tetraodon nigroviridis (CAG02018.1)
Strongylocentrotus purpuratus (XP_796363.1)
Caenorhabditis elegans (NP_496549.1)
Brugia malayi (EDP36501.1)
Oncometopia nigricans (Q5XXS4)
Apis mellifera (XP_001120025.1)
Bombyx mori (Q1HQ77)
Aedes pseudoscutellaris (ABL74415.1)
Aedes aegypti (EAT45215.1)
Aedes albopictus (ABL74414.1)
Anopheles gambiae (XP_321482.2)
Drosophila melanogaster (O76742)
Penaeus monodon (ABB70064.1)
Schistosoma japonicum (AAP06474.1)
Nematostella vectensis (XP_001637395.1)
Aiptasia pulchella (Q6EIK7)
Capsaspora owczarzaki (NUO00064598)
Monosiga ovata (MNO00063709)
Monosiga brevicollis (MRO00012659)
Schizosaccharomyces pombe (NP_593524.1)
Schizosaccharomyces pombe (NP_596307.1)
Yarrowia lipolytica (Q6C134)
Aspergillus parasiticus (AAS92973.1)
Aspergillus clavatus (XP_001274503.1)
Aspergillus terreus (XP_001211238.1)
Neosartorya fischeri (XP_001259484.1)
Emericella nidulans (BAB88682.1)
Aspergillus nidulans (Q5BH91)
Phaeosphaeria nodorum (EAT82273.1)
Coccidioides immitis (XP_001239602.1)
Chaetomium globosum (XP_001229066.1)
Magnaporthe grisea (AAX07679.1)
Neurospora crassa (Q9C2L8)
Gibberella zeae (Q4ICB7)
Cryptococcus neoformans (XP_567629.1)
Ustilago maydis (Q4P302)
Coprinellus disseminatus (Q1WMV2)
Coprinopsis cinerea (EAU87363.1)
Coprinopsis cinerea (EAU92923.1)
Lentinula edodes (Q870G4)
Batrachochytrium dendrobatidis (BDO00188679)
Spizellomyces punctatus (SPO00093612)
Mortierella verticillata (MAO00082091)
Malawimonas californiana (MCO00071097)
Malawimonas jakobiformis (MJO00255273)
Acanthamoeba castellanii (ACO00142494/500)
Mastigamoeba balamuthi (MBO00138492)
Hyperamoeba dachnaya (HDO00016682)
Dictyostelium discoideum (P36411)
Guillardia theta (GTO00074124)
Reclinomonas americana (RAO00000800/129827)
Histiona aroides (HAO00040659)
Jakoba libera (JLO00020240/5)
Jakoba bahamensis (JBO00069268)
Trimastix pyriformis (TPO00069941)
Lotus corniculatus (Q40213)
Pisum sativum (P31022)
Cucumis sativus (Q6UNT4)
Arabidopsis thaliana (Q9XI98)
Arabidopsis thaliana (Q9LS94)
Solanum tuberosum (ABB02623.1)
Nicotiana tabacum (Q40527)
Oryza sativa (Q84V98)
Oryza sativa (Q75IJ1)
Pennisetum ciliare (Q40787)
Mesembryanthemum crystallinum (P93267)
Lotus corniculatus (Q40214)
Capsicum annuum (ABK15561.1)
Nicotiana tabacum (Q40526)
Prunus armeniaca (O24461)
Arabidopsis thaliana (Q9C820)
Arabidopsis thaliana (Q9LW76)
Gossypium hirsutum (Q9XER8)
Oryza sativa (Q5N7Z9)
Pennisetum glaucum (Q5MGQ9)
Arabidopsis thaliana (Q948K6)
Arabidopsis thaliana (Q9SJ11)
Medicago truncatula (ABE87705.1)
Arabidopsis thaliana (O04157)
Glycine max (Q43463)
Vigna aconitifolia (Q41640)
Lotus corniculatus (Q40211)
Lotus corniculatus (Q40212)
Nicotiana tabacum (Q40528)
Arabidopsis thaliana (Q948K8)
Hordeum vulgare (Q3S835)
Oryza sativa (NP_001056165.1)
Oryza sativa (Q5JLU1)
Batrachochytrium dendrobatidis (BDO00029415)
Chlamydomonas reinhardtii (Q39573)
Volvox carteri (P36864)
Polytomella parva (POO00087893)
Euglena gracilis (ELO00251522)
Ostreococcus tauri (CAL51588.1)
Xenopus tropicalis (NP_989167.1)
Danio rerio (NP_001013496.1)
Rab7c
Homo sapiens (Q96AH8)
Pan troglodytes (XP_528612.2)
Macaca mulatta (XP_001082679.1)
Rattus norvegicus (XP_577269.2)
Mus musculus (Q8VEA8)
Canis familiaris (XP_848668.1)
Bos taurus (NP_001071481.1)
Gallus gallus (XP_001235016.1)
Xenopus laevis (AAI29678.1)
Xenopus laevis (AAH73279.1)
Rab7b
Entamoeba histolytica (XP_650041.1)
Trichomonas vaginalis (EAY23401.1)
Trichomonas vaginalis (Q4G2D1)
Trichomonas vaginalis (XP_001301291.1)
Trichomonas vaginalis (XP_001307759.1)
Trichomonas vaginalis (XP_001320899.1)
Trichomonas vaginalis (Q4G2D0)
Trichomonas vaginalis (Q4G2D2)
Streblomastix strix (SSO00103932)
Trimastix pyriformis (TPO00038479)
Blastocystis hominis (BHO00050329)
Dictyostelium discoideum (XP_001134507.1)
Entamoeba histolytica (Q6AW63)
Acanthamoeba castellanii (ACO00058924/3)
Entamoeba histolytica (Q6AW61)
Entamoeba histolytica (Q6AW62)
Entamoeba histolytica (Q6AW65)
Entamoeba histolytica (Q6AW66)
Entamoeba histolytica (Q6AW64)
Entamoeba histolytica (Q5NT44)
Entamoeba histolytica (Q6AW67)
Homo sapiens (NP_057454.1)
Bos taurus (NP_001069009.1)
Canis familiaris (XP_538124.1)
Rattus norvegicus (XP_346352.1)
Mus musculus (NP_795945.1)
Gallus gallus (XP_001232252.1)
Xenopus tropicalis (NP_001072679.1)
Tetraodon nigroviridis (CAF99110.1)
Danio rerio (XP_001339006.1)
Gallus gallus (NP_001008678.1)
Bos taurus (NP_001069006.1)
Mus musculus (NP_062747.1)
Rattus norvegicus (Q99P75)
Homo sapiens (P51151)
Canis familiaris (XP_537956.1)
Cercopithecus aethiops (AAT48712.1)
Xenopus laevis (AAH72859.1)
Xenopus laevis (AAH68782.1)
Xenopus tropicalis (NP_001072637.1)
Danio rerio (NP_001038931.1)
Aedes aegypti (EAT39541.1)
Anopheles gambiae (XP_318959.2)
Drosophila melanogaster (NP_609966.1)
Drosophila pseudoobscura (EAL33329.1)
Tribolium castaneum (XP_970524.1)
Apis mellifera (XP_394445.3)
Strongylocentrotus purpuratus (XP_782831.1)
Monosiga brevicollis (XP_001749566.1)
Nematostella vectensis (XP_001626795.1)
Rab9
Trypanosoma cruzi (Q4E4T4)
Trypanosoma cruzi (Q9XZK0)
Leishmania braziliensis (Q8T6H9)
Leishmania major (Q9N2P5)
Trypanosoma brucei (Q3S1K6)
Theileria annulata (XP_955364.1)
Theileria parva (Q4MZ25)
Babesia bovis (Q7YZQ5)
Paramecium tetraurelia (CAI44533.1)
Paramecium aurelia (Q6DUB4)
Paramecium aurelia (Q95UJ0)
Paramecium tetraurelia (CAI44446.1)
Paramecium tetraurelia (CAI39303.1)
Tetrahymena thermophila (Q9U5G8)
Acanthamoeba castellanii (ACO00056273)
Pyrsonympha grandis (BAF36669.1)
Symbiotic protist of Reticulitermes speratus (BAF36694.1)
Symbiotic protist of Reticulitermes speratus (BAF36695.1)
Entamoeba histolytica (Q9BLE8)
Oxytricha trifallax (OTO00019102/06/11/12/15)
Cryptosporidium parvum (XP_001388379.1)
Plasmodium berghei (XP_675019.1)
Plasmodium chabaudi (XP_742861.1)
Plasmodium yoelii (Q7RNJ2)
Plasmodium falciparum (Q9NFG0)
Plasmodium falciparum (NP_704574.1)
Candida albicans SC5314 (XP_714835.1)
Debaryomyces hansenii (XP_458573.1)
Kluyveromyces lactis (XP_453125.)
Ashbya gossypii (NP_983406.1)
Saccharomyces cerevisiae (NP_013713.1)
Candida glabrata (XP_445802.1)
Acanthamoeba castellanii (ACO00086387)
Sawyeria marylandensis (SMO00053062)
Dictyostelium discoideum (Q54K69)
Homo sapiens Rab32 (Q13637)
Homo sapiens Rab38 (P57729)
Homo sapiens Rab29 (O14966)
Homo sapiens Rab23 (Q9ULC3)
other Rabs
0.3 aa
substitution/site
p > 0.95
0.90 < p ≤ 0.95
p ≤ 0.90
