# Supplementary material for: Phylogeny and evolution of Rab7 and Rab9 proteins
Source: BMC Evol Biol. 2009 May 14;9:101. doi: 10.1186/1471-2148-9-101 (PMC2693434; doi:10.1186/1471-2148-9-101)
Supplement: Additional file 6 — Expression analyses. Tables containing results of expression analyses of genes coding for Rab7 and Rab9 proteins. [file 1471-2148-9-101-S6.doc]

Table S2. Number of ESTs for two Rab9 isoforms in different organisms. Statistically significant (p < 0.05) differences between compared fractions of ESTs are denoted in **bold**.

| organism | Rab9a | | Rab9b | | total number of ESTs | p_BH_ |
| --- | --- | --- | --- | --- | --- | --- |
|  | UniGene Acc. nr | number of ESTs | UniGene Acc. nr | number of ESTs |  |  |
| *Bos taurus* | Bt.3243 | 64 | Bt.25034 | 10 | 932239 | **1.4·10^-9^** |
| *Canis familiaris* | Cfa.316 | 10 | Cfa.10479 | 3 | 280223 | 0.11 |
| *Gallus gallus* | Gga.21374 | 34 | Gga.39678 | 32 | 368419 | 0.90 |
| *Homo sapiens* | Hs.495704 | 174 | Hs.522736 | 70 | 5800250 | **1.1·10^-10^** |
| *Mus musculus* | Mm.25306 | 263 | Mm.44557 | 125 | 6891596 | **1.4·10^-11^** |
| *Rattus norvegicus* | Rn.35289 | 13 | Rn.99440 | 5 | 511505 | 0.11 |
| *Sus scrofa* | Ssc.1863 | 70 | Ssc.61018 | 1 | 1021993 | **5.6·10^-15^** |
| *Xenopus tropicalis* | Str.7043 | 31 | Str.53522 | 13 | 1082570 | **0.017** |

Acc. nr – accession number

p_BH_ - p-value corrected by the Benjamini-Hochberg procedure

Table S3. Number of ESTs for two Rab7 isoforms in different organisms. All the differences between compared fractions of ESTs are statistically significant (p < 0.05).

| organism | Rab7a | | Rab7b | | total number of ESTs | p_BH_ |
| --- | --- | --- | --- | --- | --- | --- |
|  | UniGene Acc. nr | number of ESTs | UniGene Acc. nr | number of ESTs |  |  |
| *Bos taurus* | Bt.41314 | 202 | Bt.11413 | 34 | 932239 | **2.4·10^-27^** |
| *Canis familiaris* | Cfa.45048 | 12 | Cfa.41102 | 2 | 280223 | **0.016** |
| *Gallus gallus* | Gga.5872 | 74 | Gga.35339 | 2 | 368419 | **4.6·10^-16^** |
| *Homo sapiens* | Hs.15738 | 2008 | Hs.534612 | 109 | 5800250 | **0** |
| *Mus musculus* | Mm.333233 | 843 | Mm.44508 | 63 | 3368582 | **2.1·10^-147^** |
| *Rattus norvegicus* | Rn.1425 | 134 | Rn.140691 | 4 | 511505 | **0** |

Acc. nr – accession number

p_BH_ - p-value corrected by the Benjamini-Hochberg procedure

Table S4. Number of ESTs for different Rab7 isoforms/copies in vertebrate species.

| organism | isoform (Protein Acc. nr) | UniGene Acc. nr | number of ESTs | total number of ESTs |
| --- | --- | --- | --- | --- |
| *Danio rerio* | Rab7a (Q7T2C6) | Dr.12260 | 24 | 739876 |
|  | Rab7a (NP_001002178.1) | Dr.32183 | 13 |  |
|  | Rab7a (NP_001005591.1) | Dr.45927 | 5 |  |
|  | Rab7c (NP_001013496.1) | Dr.88418 | 9 |  |
| *Xenopus laevis* | Rab7a (Q6DCV5) | Xl.9474 | 14 | 370602 |
|  | Rab7b (AAH73279.1) | Xl.47621 | 5 |  |
|  | Rab7b (AAI29678.1) | Xl.56767 | 2 |  |
| *Xenopus tropicalis* | Rab7a (Q66JI1) | Str.4511 | 188 | 1082570 |
|  | Rab7b (NP_001096381.1) | Str.21291 | 10 |  |
|  | Rab7c (NP_989167.1) | Str.53162 | 11 |  |

Acc. nr – accession number

Statistically significant differences between EST fractions were found for pairs: Dr.45927 and Dr.12260 (p = 0.005), Dr.88418 and Dr.12260 (p = 0.04), Xl.9474 and Xl.56767 (p = 0.018), Str.4511 and Str.53162 (p = 1.5·10^-35^), Str.21291 and Str.4511 (p = 8.2·10^-36^).

Table S5. Number of ESTs for different Rab7 isoforms/copies in plant species.

| organism | lineage number in Fig. 1B and Fig. 6 (Protein Acc. nr) | UniGene Acc. nr | number of ESTs | total number of ESTs |
| --- | --- | --- | --- | --- |
| *Arabidopsis thaliana* | lineage III (Q9XI98) | At.19280 | 19 | 107493 |
|  | lineage III (Q9LS94) | At.24625 | 13 |  |
|  | lineage II (Q9LW76) | At.7607 | 6 |  |
|  | lineage II (Q9C820) | At.22905 | 1 |  |
|  | lineage I (Q948K8) | At.26414 | 2 |  |
|  | lineage I (Q9SJ11) | At.14268 | 1 |  |
| *Oryza sativa japonica* | lineage III (Q75IJ1) | Os.12305 | 130 | 837877 |
|  | lineage II (Q5N7Z9) | Os.11710 | 55 |  |
|  | lineage I (Q5JLU1) | Os.40181 | 21 |  |
|  | lineage I (NP_001056165) | Os.54425 | 2 |  |
| *Nicotiana tabacum* | lineage III (Q40527) | Nta.1401 | 11 | 56372 |
|  | lineage II (Q40526) | Nta.3509 | 2 |  |
|  | lineage I (Q40528) | Nta.467 | 2 |  |

Acc. nr – accession number

The differences were statistically significant (p < 0.035) for all comparisons of each of two *A. thaliana* genes from the lineage III with genes from other lineages with the exception to the pair: At.24625 and At.7607 (p = 0.25). All pair-wise comparisons of *O. sativa* EST fractions were also significant with p < 0.00018. Similarly, the *N. tabacum* gene from the lineage III is significantly more expressed than genes from other lineages (p = 0.04).
