# Supplementary material for: Phylogeny and evolution of Rab7 and Rab9 proteins
Source: BMC Evol Biol. 2009 May 14;9:101. doi: 10.1186/1471-2148-9-101 (PMC2693434; doi:10.1186/1471-2148-9-101)
Supplement: Additional file 7 — Sequence misannotation. List and description of sequences wrongly annotated as Rab7 or Rab9 proteins. They actually belong to other Rab subfamilies. [file 1471-2148-9-101-S7.doc]

**Sequence misannotation**

During the collection of sequences we found some of them to be misannotated because they showed domains specific to other subfamilies and did not cluster with reliable Rab7 or Rab9 sequences (Table S6). Moreover, many sequences are described as Rab 7-like (Rab-7L1) or just Rab7 proteins which is misleading since they actually belong to Rab38/Rab32 subfamily (cd04107). An interesting case is the sequence XP_001320899.1 from *Trichomonas vaginalis* that is 1044 amino acid residues long, whereas typical Rab7 and Rab9 proteins are ~ 200 residues in length. Three regions were annotated in this sequence: characteristic of Rab7 subfamily, characteristic of Rab4 subfamily and incomplete Smc domain characteristic of chromosome segregation ATPases engaged in cell division and chromosome partitioning. Since this record has a provisional status and has not been subject to final NCBI review, it could be an artifact resulting from automatic computational gene prediction and/or genomic sequence errors. Probably this record contains sequences of three proteins encoded by three neighboring separate genes tandemly arranged on chromosome.

Table S6. Sequences that are currently annotated as Rab7 proteins but they actually belong to other Rab subfamilies.

| Accession number | Organism | Subfamily membership  according to CDD search |
| --- | --- | --- |
| Q2F5M5 | *Bombyx mori* | Rab4 (cd04113) |
| Q43462, AAA34003.1 | *Glycine max* | Rab1/Ypt1 (cd01869) |
| Q4QH97, CAJ03022.1 | *Leishmania major* | Rab11_like (cd01868) |
| P90642, AAX70580.1, AAZ13227.1, XP_824785.1 | *Trypanosoma brucei* | Rab11_like (cd01868) |
| Q4CUF4 | *Trypanosoma cruzi* | Rab11_like (cd01868) |
| Q4CV37 | *Trypanosoma cruzi* | Rab11_like (cd01868) |
